# Clustering analysis of microRNA and mRNA expression data from TCGA using maximum edge-weighted matching algorithms

**DOI:** 10.1186/s12920-019-0562-z

**Published:** 2019-08-05

**Authors:** Lizhong Ding, Zheyun Feng, Yongsheng Bai

**Affiliations:** 1 Department of Biology, Indiana State University, Terre Haute, IN 47809 USA; 2 Department of Mathematics and Computer Science, Indiana State University, Terre Haute, IN 47809 USA; 3 Department of Internal Medicine, University of Michigan, Ann Arbor, MI 48105 USA

**Keywords:** Cancer, miRNAs and mRNAs, Gene regulation, BRCA, TCGA, Bipartite graph, Graph partitioning, Hungarian algorithm, Blossom algorithm, Clustering

## Abstract

**Background:**

microRNA (miRNA) is a short RNA (~ 22 nt) that regulates gene expression at the posttranscriptional level. Aberration of miRNA expressions could affect their targeting mRNAs involved in cancer-related signaling pathways. We conduct clustering analysis of miRNA and mRNA using expression data from the Cancer Genome Atlas (TCGA). We combine the Hungarian algorithm and blossom algorithm in graph theory. Data analysis is done using programming language R and Python.

**Methods:**

We first quantify edge-weights of the miRNA-mRNA pairs by combining their expression correlation coefficient in tumor (T_CC) and correlation coefficient in normal (N_CC). We thereby introduce a bipartite graph partition procedure to identify cluster candidates. Specifically, we propose six weight formulas to quantify the change of miRNA-mRNA expression T_CC relative to N_CC, and apply the traditional hierarchical clustering to subjectively evaluate the different weight formulas of miRNA-mRNA pairs. Among these six different weight formulas, we choose the optimal one, which we define as the integrated mean value weights, to represent the connections between miRNA and mRNAs. Then the Hungarian algorithm and the blossom algorithm are employed on the miRNA-mRNA bipartite graph to passively determine the clusters. The combination of Hungarian and the blossom algorithms is dubbed maximum weighted merger method (MWMM).

**Results:**

MWMM identifies clusters of different sizes that meet the mathematical criterion that internal connections inside a cluster are relatively denser than external connections outside the cluster and biological criterion that the intra-cluster Gene Ontology (GO) term similarities are larger than the inter-cluster GO term similarities. MWMM is developed using breast invasive carcinoma (BRCA) as training data set, but can also applies to other cancer type data sets. MWMM shows advantage in GO term similarity in most cancer types, when compared to other algorithms.

**Conclusions:**

miRNAs and mRNAs that are likely to be affected by common underlying causal factors in cancer can be clustered by MWMM approach and potentially be used as candidate biomarkers for different cancer types and provide clues for targets of precision medicine in cancer treatment.

**Electronic supplementary material:**

The online version of this article (10.1186/s12920-019-0562-z) contains supplementary material, which is available to authorized users.

## Background

Cancer is an abnormal growth of cells, which divide without control and spread into surrounding tissue. According to the website of the Cancer Statistics Center from the American Cancer Society (https://cancerstatisticscenter.cancer.org/#!/), in 2017 in the U.S., there were an estimated 1,688,780 new cancer cases and 600,920 cancer deaths. Cancer is a genetic disease caused by alterations of genes that control the cell behavior, like cell growth and division. Genetic, transcriptional as well as other alterations can be comprehensively identified from next generation sequencing (NGS) data of samples collected from tumorous tissue and normal adjacent tissue in the same patients suffering from a specific type of cancer. Those data are accumulated and organized by different projects such as International Cancer Genome Consortium (ICGC) [1], Encyclopedia of DNA Elements (ENCODE) [2], and the Cancer Genome Atlas (TCGA) [3]. The TCGA project was initiated in 2006 to develop a publicly-accessible infrastructure of data. TCGA finalized tissue collection with matched tumor and normal tissues from 11,000 patients with 33 cancer types and subtypes, including 10 rare types of cancer. TCGA data has been used to characterize key genomic changes, find novel mutations, define intrinsic tumor types, discover similarities and differences across cancer types, reveal therapy resistance mechanisms, and collect tumor evolution evidence [3].

microRNA (miRNA) is a very short RNA (~ 22 nt) that can regulate gene expression at the post-transcriptional level [4]. Mainly from either intronic or intergenic regions of noncoding or coding genes [5, 6], miRNAs are transcribed primarily by RNA polymerase II to be parts of longer primary miRNA (pri–miRNA) transcripts that are capped, spliced, and polyadenylated [7, 8]. In the nucleus, pri–miRNA is processed, by the Microprocessor complex that consists of the RNase III enzyme Drosha and its cofactor DGCR8, to produce precursor miRNA hairpin (pre–miRNA). The resulting pre–miRNA is then exported to the cytoplasm and cleaved by Dicer to produce miRNA:miRNA duplex. Then the functional miRNA strand and Argonaute (AGO2) proteins are incorporated into the RNA–induced silencing complex (RISC) [9]. The base pairing between miRNA and mRNA relies on the seed region, about 2–8 nt in an miRNA, which functions as a part in the RISC, bound to the complementary region in the 3′ UTR of its target mRNA [10]. The miRNA guides RISC to silence the target mRNAs by means of mRNA cleavage, translational repression, or deadenylation [11].

Regulation of the miRNA and mRNA network is complex. A single miRNA can target many mRNAs, while many miRNAs are able to cooperatively target a single mRNA. This allows for fine-tuned gene expression regulation [12]. The cooperativity within some miRNA families or genomic clusters that target the same mRNAs is likely to be mainly additive [10]. miRNA also has sponge function for mRNA. When one of the mRNAs targeted by a specific miRNA change its expression level, the specific miRNA will redistribute and alter the protein translation of several transcripts. [13]. Thereby, considering these complexity, the observed expression correlation coefficient of a particular miRNA-mRNA targeting pair can range from − 1 to 1, not always negative, even if the miRNA-mRNA has predicted or experimentally validated targeting relationships. The aberration of miRNA expression could affect a large number of mRNAs and cancer-related signaling pathways [14]. Some previous studies discovering and explaining this complexity in cancer are summarized as follows:

In a breast cancer study, miR-183 was experimentally proven to directly target the 3′-UTR of its target gene RAB21, by co-transfecting the luciferase reporters with 33 bp of the predicted target regions. The miR-183/− 96–182 genomic cluster also has transcription factors HSF2 and ZEB1 that are experimentally validated to bind to the upstream of the TSS region of the miR-183/− 96–182. Nevertheless, analyzing the 508 clinical samples from TCGA data, the correlations between miR-183/− 96/− 182 cluster miRNAs and their target/regulators do not exhibit simply positive or negative correlations. Experimentally verified direct correlation between miR-183 and the expressions of RAB21 could not be found based on the TCGA data analysis. But some interesting correlations between them in different subtypes were found [15], indicating the clue of solving the miRNA-mRNA network complexity by grouping the subtypes of the cancer types.

In a study of ovarian cancer, it was found that the miRNA-mRNA pair hsa-miR-140-3p/RAD51AP1, was negatively correlated in both normal and tumorous samples with downregulated miRNA and upregulated mRNA expression values in tumor relative to normal samples, suggesting the expressional dysregulation of a direct miRNA-mRNA interaction mechanism. However, some miRNA-mRNA pairs were positively or negatively correlated in the tumor samples, but not in the normal samples, implying that the miRNAs de novo gain functions in tumor. Some miRNA-mRNA pairs show correlations in normal samples but not in tumor samples, implying that the miRNA de novo lose functions in tumors. Intriguingly, the miRNA-mRNA pairs that are positively correlated in both tumor and normal samples were identified, suggesting potential indirect pathways or intermediate regulatory mechanisms in tumorigenesis [16].

There are bioinformatics tools clustering the miRNA-mRNA interaction network [17]. Some tools are based on miRNA-mRNA expressional correlation coefficients calculated from NGS expression data [18]. Clustering results can be enhanced or filtered by integrated analysis of known or predicted miRNA-mRNA targeting information [19]. For example, MAGIA2 utilizes negative expressional correlation coefficients between miRNA and mRNA across many matched or unmatched samples [19]. However, MAGIA2 neglects the situation that the miRNAs that have positive correlation coefficients also play a role in the regulatory network. miRMAP studies both significant negative and positive correlations between miRNA and mRNA; its bicluster analysis of miRNA-mRNA bipartite graph provides insights into how modules of miRNAs regulate groups of functionally related mRNAs [10]. However, miRMAP only considered tumor condition. Thereby it lacks the view of the correlation coefficient changes between normal and tumor tissues. MMiRNA-Viewer visualizes altered expressional correlation coefficients of miRNA and mRNA in both tumor and normal; the correlation coefficient of a miRNA-mRNA pair could be the same or inverted in sign in tumor compared to normal [18]. However, the connections between miRNA and mRNA are not combined together to quantify the miRNA-mRNA correlation coefficient changes from normal to tumor.

Although Jansson and Lund explained the potential mechanisms of how a target mRNA may become uncoupled from its targeted miRNA [14], the factors inverting the miRNA-mRNA expression correlation coefficient from normal to tumor are still unclear, indicating the complex direct and indirect regulation of the miRNA and mRNA network. In this study, we proposed six weight formulas to quantify the change of miRNA-mRNA expressional correlation coefficients in tumor relative to in normal. We used the traditional hierarchical clustering algorithm to evaluate different formula weights of miRNA-mRNA pairs and chose the integrated mean value weight. Then, we developed a novel bioinformatics pipeline called maximum weighted merger method (MWMM) based on objective optimization algorithms, namely the Hungarian and blossom algorithm, to cluster the miRNA–mRNA pairs. We hypothesized that the miRNA-mRNA pairs with higher weights, if properly clustered together, are more likely to be intensely affected by common causal factors in the complex direct and indirect network.

## Methods

**Fig. 1:**
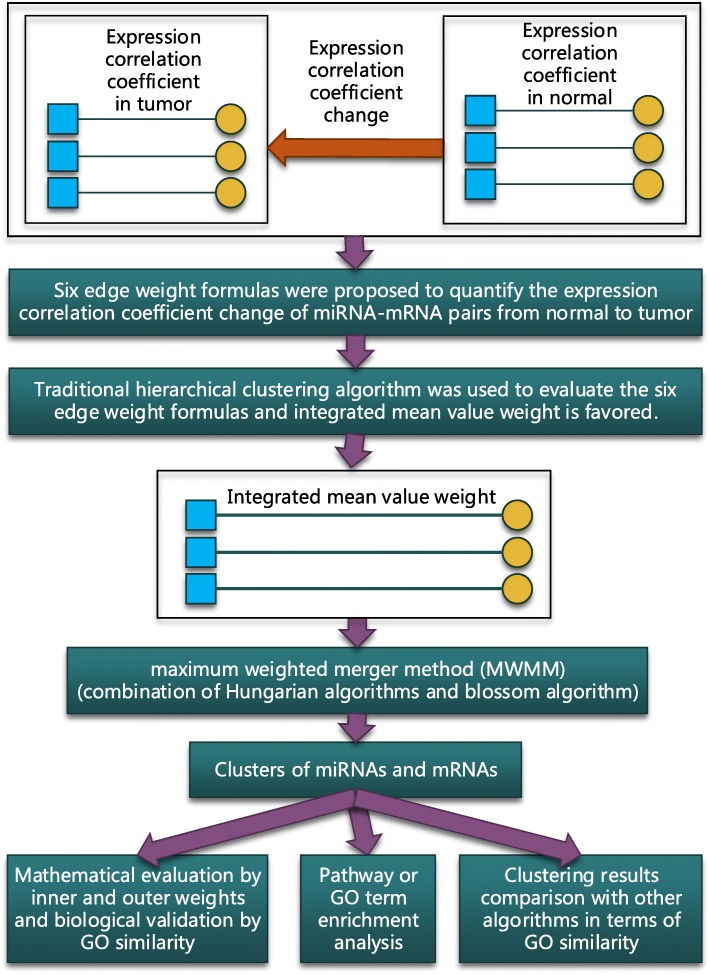
Conceptual steps of the whole methodology of pipeline. Blue squares denote miRNA. Yellow circles denote mRNAs

This study focused on the expression correlation coefficient changes of miRNA-mRNA pairs that were inverse from in normal to in tumor. Six edge weight formulas, which were proposed to simultaneously quantify the changes, were evaluated using the subjective traditional hierarchical clustering algorithm. After evaluation, integrated mean value weight was used to quantify the changes. Then, a maximum weighted merger method (MWMM) pipeline that consists of continuous iterations of Hungarian algorithm and several rounds of blossom algorithm was used to passively cluster the miRNA-mRNA pairs based on the maximum weighted edge matching in the bipartite graph and general graph. The clustered miRNA-mRNA pairs were validated mathematically by the clustering criteria that the inner weights of the clusters are larger than the outer weights of the clusters and biologically by the criteria that intra-cluster’s average GO term similarity distance scores are larger than the inter-cluster’s. Then the genes in clusters were enriched via functional analysis like KEGG pathway or GO term. Finally, the effectiveness of MWMM was tested by applying the MWMM approach to other 14 cancer types and comparing to other six clustering algorithms in terms of GO term similarity distance scores. The methodology is illustrated in Fig. 1.

### Source data of BRCA from TCGA

The invasive breast carcinoma (BRCA) NGS expression data of miRNA and mRNA in tumor and in normal were downloaded from the TCGA data portal. BRCA data set has 863 samples consisting of 87 normal samples and 776 tumor samples. The number of samples in BRCA and other TCGA data sets used in this study are described in the first table in [18]. We processed the downloaded BRCA data set following the same procedure described in [20], and got four expression matrices across samples, including miRNA expression in tumor, miRNA expression in normal, mRNA expression in tumor, and mRNA expression in normal. The expression matrices involve 1046 miRNA and 20,531 mRNA in both tumor and normal samples. The row names of the expression matrices are miRNA names or gene names. The column names of the expression matrices are sample names.

Then we used MMiRNA-Tar [20] that takes four expression matrices as input to calculate the expression correlation coefficient (Pearson correlation coefficient) across samples for each possible miRNA-mRNA pair combination. The resultant miRNA-mRNA pairs in tumor and normal are filtered following three cutoff criteria: false discovery rates (FDR) are ≤ 0.1, the correlation coefficients in tumor are opposite to in normal samples, and target predictions are supported by at least one of three target prediction databases: TargetProfiler [21], TargetScan [22], and miRanda [23]. Eventually we end up with 20,661 pairs of miRNA and mRNAs with their expression correlation coefficients in tumor (T_CC) and correlation coefficients in normal (N_CC) calculated and organized into a table for downstream analysis. The table is exemplified in Table 1.

**Table 1:** Calculated miRNA and mRNA expression expressional correlation coefficients in tumor and normal tissue of BRCA. The first 10 miRNA-mRNA pairs of the table that has 20,661 pairs are listed as an example

mRNA	microRNA	T_CC	N_CC
*OBFC1*	*hsa-mir-383*	−0.092	0.271
*SHROOM2*	*hsa-mir-130a*	−0.098	0.442
*GABBR2*	*hsa-mir-452*	0.142	−0.376
*ZNF90*	*hsa-mir-452*	0.139	−0.365
*GIGYF1*	*hsa-mir-3653*	−0.192	0.281
*MICALL1*	*hsa-mir-375*	−0.269	0.305
*ZNF552*	*hsa-mir-30e*	−0.142	0.279
*MT2A*	*hsa-mir-744*	−0.089	0.341
*ISG20*	*hsa-mir-215*	0.110	−0.373
*PJA1*	*hsa-mir-204*	0.205	−0.283

### Edge-weighted bipartite graph model

**Fig. 2:**
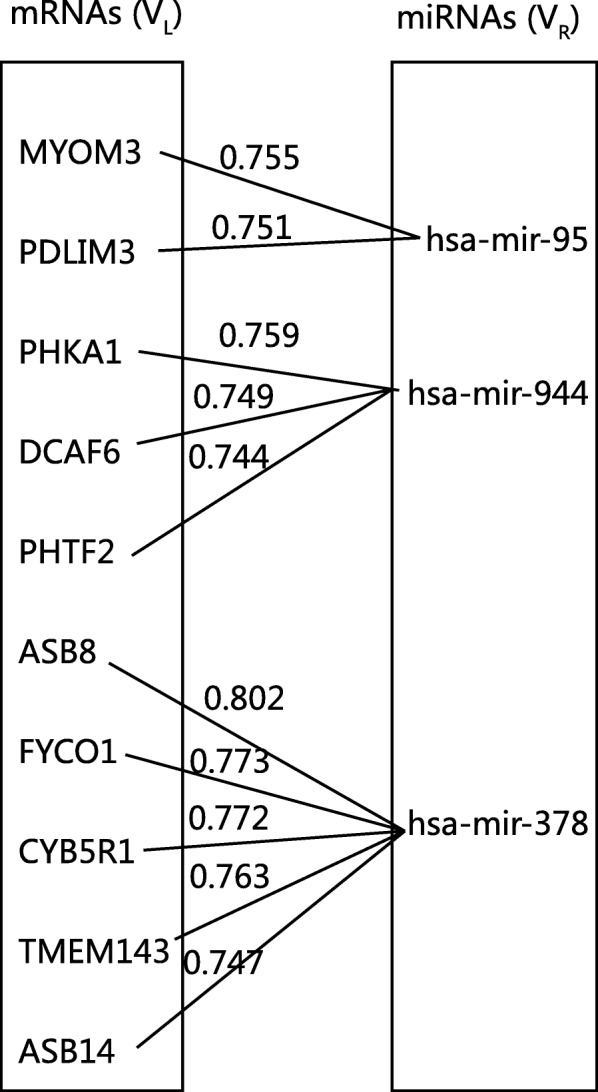
An example of a miRNA-mRNA bipartite graph. The miRNA-mRNA pairs are represented by an edge-weighted bipartite graph. The pairs of miRNA-mRNA with the 10 largest integrated mean value weights (described in the next subsection) are listed as an example in a railroad display of the bipartite graph

A simple graph is defined as *G* = (*V*, *E*), where *V*(*G*) or *V* denotes a set of vertices, and *E*(*G*) or *E* denotes a set of edges. *E* is 2-element subsets of V. An edge is associated with two vertices. w(*e*) is defined as edge weight for each edge. In this study, the miRNA-mRNA interaction network is visualized as an edge-weighted bipartite graph *G* = (*V*, *E*), where *V* consists of vertices of mRNAs (*V*_L_) and miRNAs (*V*_R_), i.e., *V* = (*V*_L_ + *V*_R_) and *E* represents the weighted edges between the mRNA and miRNA vertices. Let i be the vertex subscript in the *V*_L_ and j be the vertex subscript in the *V*_R_. Then v_i_v_j_ is the connection between v_i_ and v_j_, namely the edge connecting a vertex in *V*_L_ to a vertex in V_R_. An example of a miRNA-mRNA bipartite graph is shown in Fig. 2. The edge list denoting the bipartite graph is shown in Table 2.

**Table 2:** An example of edge list denoting a miRNA-mRNA bipartite graph. The edge list has three columns: miRNA vertex, mRNA vertex, and their edge weight. The pairs of miRNA-mRNA with the 10 largest integrated mean value weights (described in the next subsection) are listed as an example

mRNA	microRNA	weight
*ASB8*	*hsa-mir-378*	0.802
*FYCO1*	*hsa-mir-378*	0.773
*CYB5R1*	*hsa-mir-378*	0.772
*TMEM143*	*hsa-mir-378*	0.763
*PHKA1*	*hsa-mir-944*	0.759
*MYOM3*	*hsa-mir-95*	0.755
*PDLIM3*	*hsa-mir-95*	0.751
*DCAF6*	*hsa-mir-944*	0.749
*ASB14*	*hsa-mir-378*	0.747
*PHTF2*	*hsa-mir-944*	0.744

### Edge weight calculation

We combine T_CC and N_CC simultaneously to quantify the connections or weighted edges between miRNA and mRNA vertices. The connections reflect the intensity of inversion of miRNA-mRNA expression correlation coefficients from in normal to in tumor. The formulas of calculating edge weights are described as follows.

We propose 6 types of edge weights that consider information of T_CC and N_CC simultaneously to measure the connection of the edge *v*_*i*_*v*_*j*_, in the case of BRCA, for 1 ≤ i ≤ 312 and 1 ≤ j ≤ 7874. Based on the foregoing three cutoff criteria, the number of selected miRNAs is 312 and the number of selected mRNAs is 7874.

**Fig. 3:**
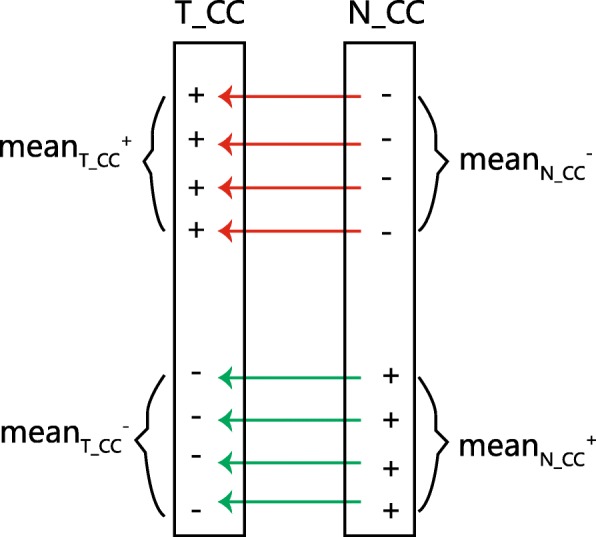
Definition of means of four subsets in T_CC and N_CC. The red arrows denote that the correlation coefficients are converted from negative in normal to positive in tumor. The green arrows denote that the correlation coefficients are converted from positive in normal to negative in tumor

The miRNA-mRNA expression correlation coefficients are separated into two classes based on their parity change, as shown in Fig. 3. One class has N_CC >  0 and T_CC <  0, i.e., the correlation coefficients are converted from positive in normal to negative in tumor. The other class has N_CC <  0 and T_CC >  0, i.e., the correlation coefficients are converted from negative in normal to positive in tumor.

Intuitively, the arithmetic mean of absolute values (ie., T_CC and N_CC) is an option to quantify the inversion of their expressional correlation coefficient, namely, inversion of N_CC to T_CC for a miRNA-mRNA pair as shown in Fig. 3. However, to increase the contrast of values in the data, a coefficient can be generated for each value by dividing that value by the arithmetic mean of the data. The value is then multiplied by its coefficient, so that values larger than the arithmetic mean of the data will become larger, and values smaller than the arithmetic mean of the data will become smaller. Using notation, let the T_CC values have the arithmetic mean, *m*_*T* _ *CC*_. A specific T_CC value is denoted *x*, while $\frac{x}{m_{T\_ CC}}$ is that value’s coefficient. The new value is given by $\frac{x}{m_{T\_ CC}}\times x$, which enhances the importance of the value *x* if *x* is greater than the average *m*_*T* _ *CC*_, and weakens the importance of the value *x* if *x* is smaller than the average *m*_*T* _ *CC*_.

In our expression correlation coefficient data, the T_CC values consist of two groups: positive values and negative values. We calculate the arithmetic mean of the positive values of T_CC as ${\boldsymbol{m}}_{\boldsymbol{T}\_\boldsymbol{CC}}^{+}$ and arithmetic mean of absolute value of the negative values of T_CC as ${\boldsymbol{m}}_{\boldsymbol{T}\_\boldsymbol{CC}}^{-}$ . Similarly, we calculate the ${\boldsymbol{m}}_{\boldsymbol{N}\_\boldsymbol{CC}}^{+}$ and ${\boldsymbol{m}}_{\boldsymbol{N}\_\boldsymbol{CC}}^{-}$ for N_CC’s groups, as shown in Fig. 3. Then, the integrated mean value weight is calculated by assignments of coefficients *λ*_1_ and *λ*_2_, shown in Table 3. In such a way, we can quantify the inversion of the correlation coefficients from the positive values of N_CC, N_CC^+^, to the negative values of T_CC, T_CC^−^ (*λ*_1_), and likewise, from N_CC^−^ to T_CC^+^ (*λ*_2_) because these two sets represent different correlation change directions, as shown in Fig. 3.

**Table 3:** Coefficient of correlation coefficient in the integrated mean value weight formula

${\uplambda}_1=\frac{m_{T\_ CC}^{+}}{m_{T\_ CC}^{+}+{m}_{N\_ CC}^{-}}$	$1-{\uplambda}_1=\frac{m_{N\_ CC}^{-}}{m_{T\_ CC}^{+}+{m}_{N\_ CC}^{-}}$
${\uplambda}_2=\frac{m_{T\_ CC}^{-}}{m_{T\_ CC}^{-}+{m}_{N\_ CC}^{+}}$	$1-{\uplambda}_2=\frac{m_{N\_ CC}^{+}}{m_{T\_ CC}^{-}+{m}_{N\_ CC}^{+}}$

Based on the above-mentioned reasoning, we propose the formula of the integrated mean value weight to combine the T_CC and N_CC information simultaneously, as shown in Table 3 and Table 4. For the sake of comparison, we also propose other formulas that are common in basic mathematics, listed in Table 4. For example, all negative value weight cannot reflect the correlation coefficient changes of a miRNA-mRNA pair in tumor relative to normal, but it can act as a random formula as a comparison to see if the proposed integrated mean value weight is random. Thereby, more possibilities exist beyond these six formulas.

**Table 4:** Formulas of six proposed weights to quantify the T_CC and N_CC simultaneously

Weight name	Formula
integrated mean value weight	$$ \left\{\begin{array}{c}{\uplambda}_1\times T\_ CC+\left(1-{\uplambda}_1\right)\times \left|N\_ CC\right|,\mathrm{if}\ \mathrm{T}\_\mathrm{CC}>0\\ {}{\uplambda}_2\times \left|T\_ CC\right|+\left(1-{\uplambda}_2\right)\times N\_ CC,\mathrm{if}\ \mathrm{T}\_\mathrm{CC}<0\end{array}\right. $$
all negative value weight	$$ \left\{\begin{array}{c}\left|N\_ CC\right|,\mathrm{T}\_\mathrm{CC}>0\\ {}\left|T\_ CC\right|,\mathrm{T}\_\mathrm{CC}<0\end{array}\right. $$
all positive value weight	$$ \left\{\begin{array}{c}T\_ CC,\mathrm{T}\_\mathrm{CC}>0\\ {}N\_ CC,\mathrm{T}\_\mathrm{CC}<0\end{array}\right. $$
arithmetic mean value weight	$\frac{\left|T\_ CC\right|+\left|N\_ CC\right|}{2}$
geometric mean value weight	$\sqrt{\left|T\_ CC\right|\times \left|N\_ CC\right|}$
maximum absolute value weight	max(|*T* _ *CC*|, |*N* _ *CC*|)

For each miRNA-mRNA pair in every row of the input table exemplified in Table 1, we calculated their six different weights. For example, the first row of the input table, the OBFC1 and hsa-mir-383 pair has TCC value − 0.092 and N_CC value 0.271. The six weights of the pair are 0.225, 0.092, 0.271, 0.182, 0.158 and 0.271, respectively using weight formulas in Table 4.

### Evaluation of six edge weight formulas by traditional hierarchical clustering algorithm

**Fig. 4:**
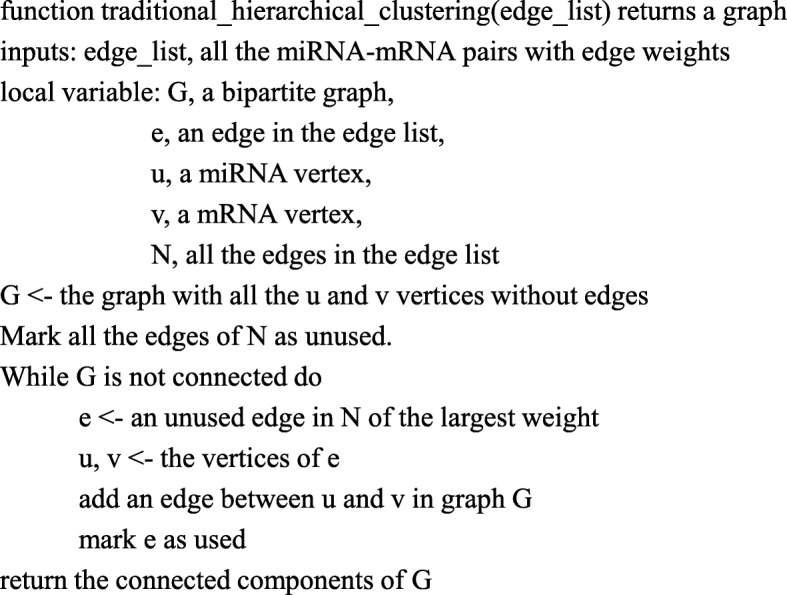
Pseudocode of traditional hierarchical clustering algorithm adapted from [24]. The algorithm is applied to a weighted edge list

The traditional hierarchical clustering algorithm [24] was performed to evaluate six edge weight formulas. A particular clustering is not defined in the traditional hierarchical clustering algorithm. Instead, a sequence of clusters is given for researchers to interpret. To run the traditional hierarchical clustering algorithm on our bipartite graph edge list, the original pseudocode in [24] is adapted and shown in Fig. 4.

To cluster the miRNA-mRNA pairs using traditional hierarchical clustering algorithm, we subjectively run n steps, namely add top-n largest weighted edges to the empty graph to obtain a new graph with n edges. First, we initialize an empty bipartite graph. In each step, from the input table of edge list, we choose a miRNA-mRNA pair if its edge weight is currently the maximum edge weights, and then add the pair to the bipartite graph; the chosen pair is removed from the input table of edge list. The process is repeated n times. n is subjectively determined by the user, rather than determined by a criterion inside the algorithm. Finally, there are n edges of miRNA-mRNA pair in the bipartite graph. The miRNA and mRNA vertices with weighed edges are visualized using igraph [25] and ggnet2 (https://briatte.github.io/ggnet/) packages in R programming language.

**Fig. 5:**
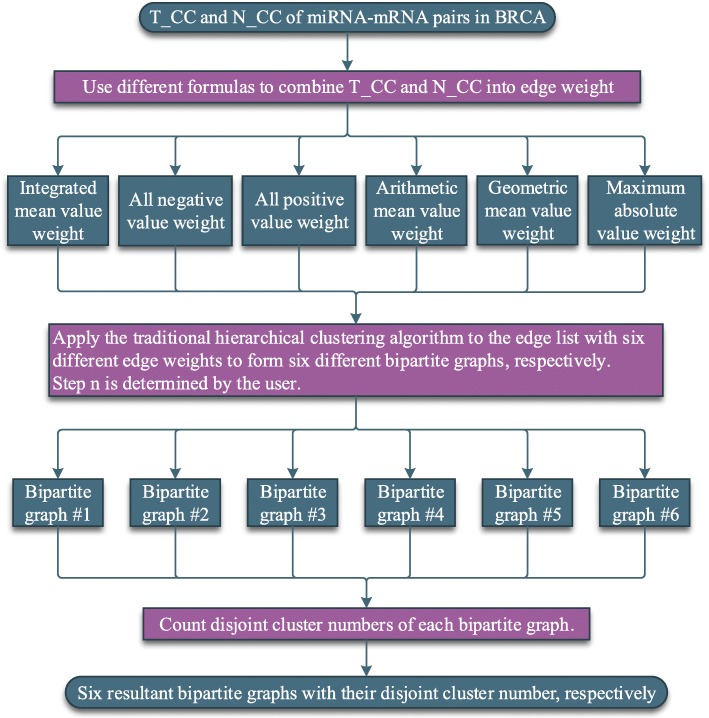
The workflow of the traditional hierarchical clustering algorithm that cluster the miRNA-mRNA pairs based on the edge weights derived from the different formulas

The traditional hierarchical clustering algorithm can be used to evaluate six edge weight formulas. Given a specific threshold of number of edges, ie., steps, in the traditional hierarchical clustering, an edge weight formula that produces smaller number of disjoint clusters suggest that more high-weighted miRNA-mRNA interactions are clustered based on this formula, so this edge weight formula are considered better than other weight formulas. Thereby, the correlation between the connected cluster number and different edges/steps using different weight formulas is studied following the workflow diagram in Fig. 5. Based on the result part, the integrated mean value weight is now adopted in the sequel.

The traditional hierarchical clustering algorithm can actively, not passively, cluster the miRNA-mRNA pairs and also can filter the top-weighted edges in the graph, because only the top weighted, namely important, edges are added to the graph. In the meantime, the smaller weight edges, which might also have biological roles are ignored. To solve this issue, we proposed an objective maximum weighted merger method (MWMM) approach that also clusters smaller weight edges and tries to achieve the global optimum instead of only clustering top-weighted edges. Thereby, traditional hierarchical clustering algorithm was only used to evaluate six edge weight formulas in this study.

### Graph partitioning of the bipartite graphs

Partitioning the graph *G* consists of dividing the vertices into clusters, such that the total weight of the edges whose end points are in different clusters is minimized. The objective of this kind of partitioning is to minimize the cut, i.e. the total weight of the edges crossing the clusters. This is equivalent to maximizing the total weight of the edges that are inside the clusters [26].

**Fig. 6:**
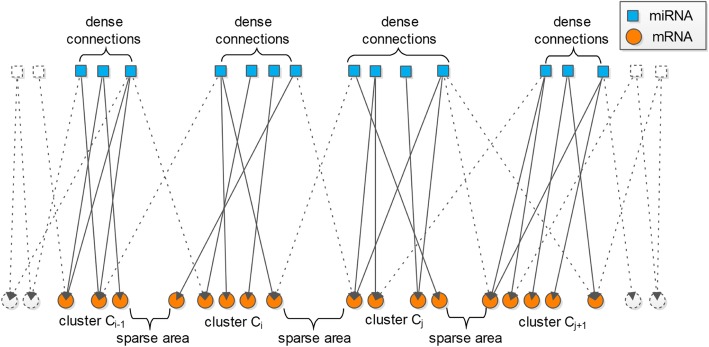
An illustration of partitioning V(G) into a number of bipartite subgraphs. The input edge-weighted graph is bipartite, i.e., V(G) = V(A)∪V(B) and all edges are linked between disjoint parts A and B, where part A represents miRNAs, with (upper) nodes denoted by squares, and part B represents mRNAs, with (lower) nodes denoted by circles

In general, a graph’s vertex set V(*G*) may be partitioned into c disjoint parts, V_1_, V_2_, …, V_c_, such that V = V_1_∪V_2_∪V_3_ … ∪V_c_. Such parts may be referred to as subgraphs, partitions, or communities, but they shall be referred to as clusters in this discussion. A cluster, with more weighted connections inside and fewer weighted connections to other clusters, indicates that the members of a cluster are more similar or linked to each other than those in the portions of the graph outside that cluster [27]. The partitioning is illustrated in Fig. 6.

### Hungarian and blossom algorithm matching in graph theory

A matching in graph theory is defined as a subset of edges such that none of the edges in the subset shares a common vertex. A maximum edge-weighted matching is a matching where the weight sum of the matched edges is as large as possible. In other words, we seek a perfect matching *M* to maximize the total weight *w*(*M*) = ∑_*e* ∈ *M*_*w*(*e*).

**Fig. 7:**
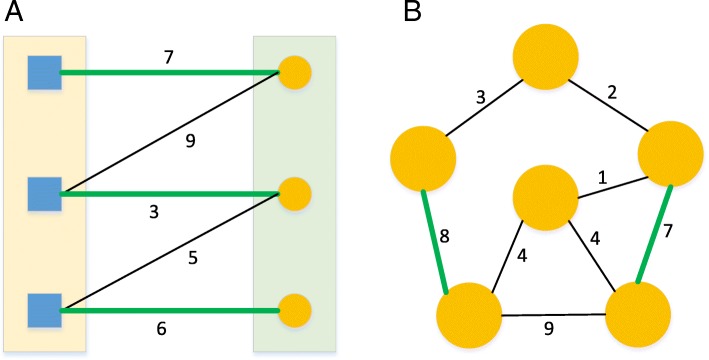
Tiny example of matching algorithms. Squares or circles are vertices. The numbers are edge weights. Green edges are edges in the maximum matching. Black edges are edges that are not in the maximum matching. (A): Hungarian algorithm that finds maximum edge-weighted matchings in a bipartite graph. (B): Blossom algorithm that finds maximum edge-weighted matchings in a general graph. The figure is adapted from https://www-m9.ma.tum.de/graph-algorithms/matchings-hungarian-method/index_en.html and http://jorisvr.nl/article/maximum-matching

The Hungarian algorithm is a combinatorial optimization algorithm used to solve the assignment problem. For example, if the performance of each of n people on each of n jobs is scored numerically, the assignment problem tries to assign people to jobs to make the sum of the scores as large as possible [28]. A tiny example of Hungarian algorithm is drawn in Fig. 7.

The Blossom Algorithm is an algorithm for finding the maximum matching in a general graph through shrinking cycles in the graph to reveal augmenting paths. The Blossom Algorithm is used to solve assignment problem, traveling salesman problem, etc. Given a general graph *G* = (*V*, *E*), the Blossom algorithm finds a matching *M* such that each vertex in *V* is incident with at most one edge in *M* and the edge weight *w*(*M*) is maximized [29]. A tiny example of blossom algorithm is drawn in Fig. 7.

### MWMM procedure

**Fig. 8:**
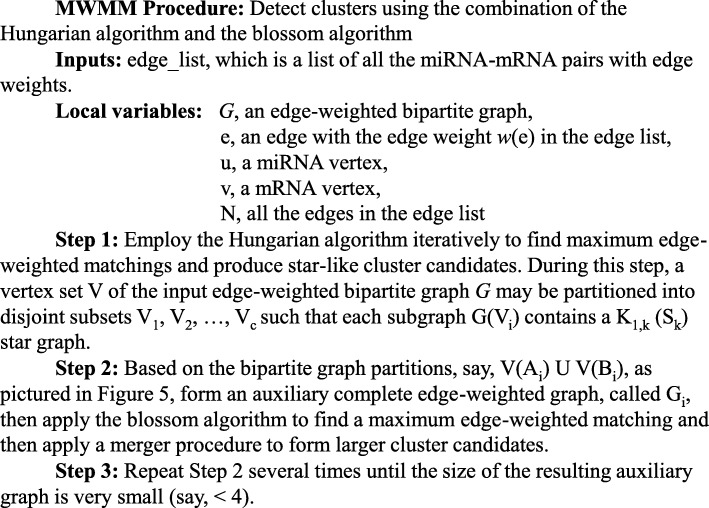
Pseudocode of the MWMM pipeline

**Fig. 9:**
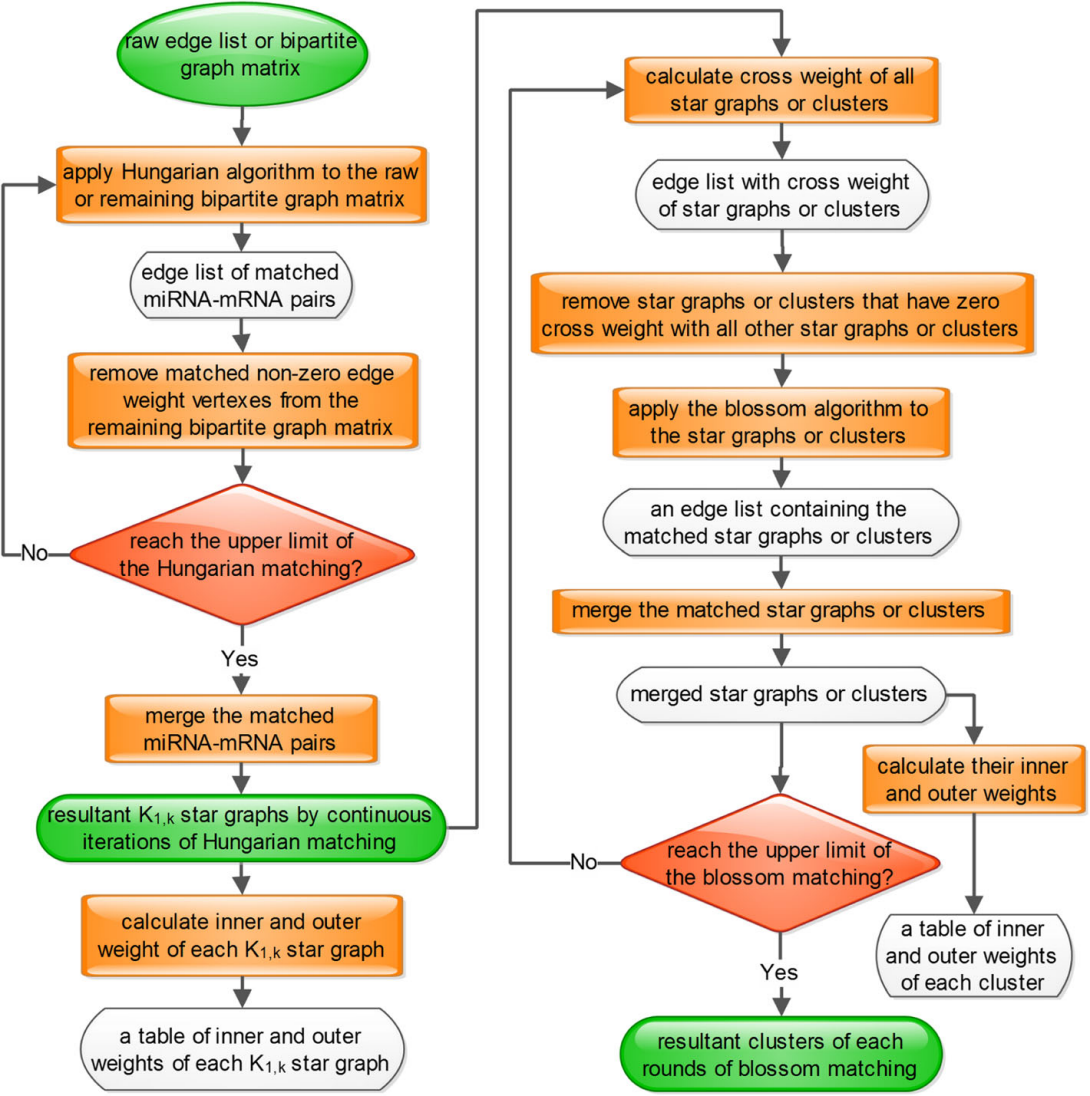
Workflow of the Hungarian-blossom clustering approach. The Hungarian algorithm stops when all the miRNA-mRNA pairs are assigned to star graphs. The blossom algorithm stops when there are at most two or three partitioning parts left. As a result, original partitions produced by Hungarian algorithm are eventually merged to several partitions by blossom algorithm

The maximum weighted merger method consists of two major stages. First, MWMM implements Hungarian algorithm to find maximum edge-weighted matchings in bipartite graphs. We iteratively constructed and combined maximum edge–weighted matchings via the Hungarian algorithm to produce disjointed star graphs, labeled from K_1,1_ to K_1,k_. Second, MWMM implements the Blossom algorithm to find maximum edge-weighted matchings in general graphs. We iteratively merge the initialized disjointed stars derived from the continuous iteration of Hungarian algorithm to form new edge–weighted clusters. The pseudocode of the MWMM pipeline is described in Fig. 8. The workflow of MWMM pipeline is depicted in Fig. 9.

Taken together, taking an edge list of edge-weighted bipartite graph, MWMM approach partition it into clusters that have higher internal connection density inside a cluster and lower external connection density outside the cluster. In other words, inner weights of a valid cluster should be greater than or equal to its outer weights. This clustering criterion is passive and objective to evaluate the quality of resulting clusters. This passive evaluation approach is different from, and better than, the subjective judgement of the cluster in the traditional hierarchical clustering approach: “clusters are in the eyes of the beholder”.

### Application of Hungarian algorithm

The Hungarian algorithm takes input of a bipartite graph matrix that has miRNAs as row names, mRNAs as column names, and edge weights as entries. This raw bipartite graph matrix is converted from the raw edge list exemplified in Table 2.

**Fig. 10:**
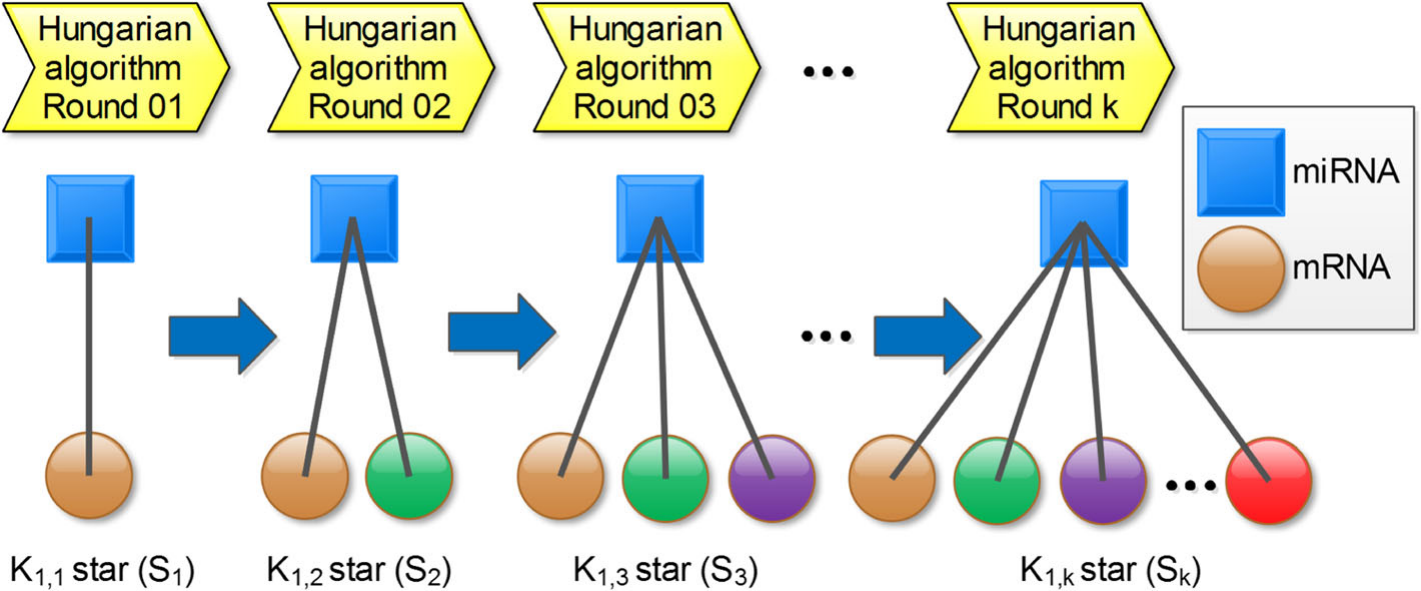
Illustrated example of constructing star-graphs. The miRNA is the internal node (denoted by a square) of a star graph and the mRNA is the leaf (denoted by a circle) of a star graph. After the first round application of the Hungarian algorithm, disjoint K_1,1_ stars (S_1_) are produced. After the second round application of the Hungarian algorithm, disjoint K_1,2_ stars (S_2_) are formed. The internal node of each K_1,2_ star (S_2_) is the same as each corresponding K_1,1_ star (S_1_), respectively. One of the leaves of each K_1,2_ star (S_2_) is derived from each corresponding K_1,1_ star (S_1_), respectively. After the third iteration of Hungarian algorithm, disjoint K_1,3_ stars (S_3_) are produced. The internal node of each K_1,3_ star (S_3_) is the same as the corresponding K_1,1_ star (S_1_) and K_1,2_ star (S_2_), respectively. Two of the leaves of each K_1,3_ star (S_3_) are derived from the corresponding K_1,2_ star (S_2_). Eventually, disjoint K_1,k_ stars (S_k_) are constructed. The zero-value-weighted edges were removed from all the star graphs

After applying each round of the Hungarian algorithm, we get an edge list of matched miRNAs and mRNAs. We remove the matched zero edge weight miRNA-mRNA pairs from the matched pairs so that the miRNAs or mRNAs in the zero edge weight matched pairs can participate in the next round of Hungarian algorithm to match their non-zero edge weight miRNA or mRNA mates, instead of being discarded. In other words, the matched zero edge weight miRNA-mRNA pairs are still in the remaining bipartite matrix for the next round of Hungarian algorithm. The matched, non-zero edge weight mRNA and miRNA pairs are used to construct star graphs shown in Fig. 10.

Before the next round of Hungarian algorithm application, the columns of matched mRNAs are removed from the remaining bipartite graph matrix, whereas the rows of matched miRNAs are usually not removed from the remaining bipartite graph matrix. However, miRNAs that have zero edge weight with all mRNAs are removed from the remaining bipartite graph matrix, when each miRNA row of the remaining bipartite graph matrix is checked before the next round of Hungarian algorithm. Since these miRNAs has been matched and stored in the internal nodes of star graphs, keeping these used miRNAs in the remaining bipartite graph matrix make the Hungarian algorithm hard to match perfectly. Finishing these processing, the next round of the Hungarian algorithm was applied to the updated remaining bipartite graph matrix.

This Hungarian algorithm was repeated until all the miRNAs and mRNAs are removed from the remaining bipartite graph matrix. Eventually, the Hungarian algorithm merging process yields 312 K_1,k_ (S_k_) star graphs. The Hungarian algorithm implementation is provided by clue [30] package in R programming language.

### Star graph construction by the Hungarian algorithm

In graph theory, a star (graph) is a complete bipartite graph that has 1 internal node and k leaves, and accordingly the star graph is named K_1,k_ star or S_k_. Note that there are 312 miRNAs and 7874 mRNAs in the raw bipartite graph. Since one miRNA can target multiple mRNAs, after continuous iterations of the Hungarian algorithm, we derived 312 merged star graphs. To facilitate programming, disjoint K_1,k_ star graphs are stored in communities object in igraph objects in R programming language. The star graph construction process is illustrated in Fig. 10.

### Cross weight of vertices denoting clusters in the auxiliary graph

**Fig. 11:**
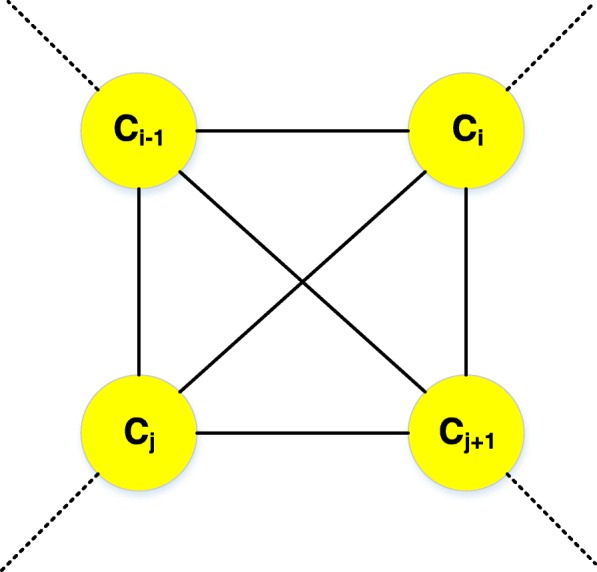
An illustration of four example vertices of C_i-1_, C_i_, C_j_, and C_j + 1_ in the auxiliary graph. Each vertex in the auxiliary graph is a (merged) cluster of miRNAs and mRNAs. The edge weights among vertices are measured by cross weights described later. The solid lines denote the connections among the four example vertices in the auxiliary graph. The likely connections between example vertices and other vertices in the auxiliary graph is represented by dotted lines

The 312 star graphs constructed by Hungarian algorithm are initial clusters that will be merged to form new clusters. Then the blossom algorithm is used to combine these star graphs or clusters. An edge-weighted auxiliary graph with 312 vertices denoting star graphs or clusters is formed by contracting each star graphs or (merged) clusters of miRNAs and mRNAs to a vertex in the auxiliary graph. For instance, we contract clusters C_i-1_, C_i_, C_j_, and C_j + 1_ in Fig. 6 to vertices C_i-1_, C_i_, C_j_, and C_j + 1_. in the auxiliary graph. The auxiliary graph is illustrated in Fig. 11.

**Fig. 12:**
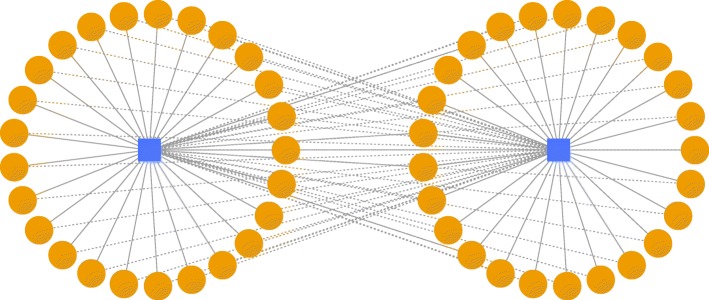
Example diagram of a cross weight calculation for two disjoint K_1,25_ star graphs (S_25_). The dashed lines denote the cross connections between the two star graphs. The solid lines denote the connections within a star graph. The weight of each connections are derived from the raw input edge list. The sum of cross connection weights (dashed lines) are calculated and then averaged by the number of vertices in the two clusters

Cross weight is defined as the sum of the weights of the connections between two star graphs or clusters averaged by the number of vertices in the two star graphs or clusters. Averaging prevents larger clusters to be merged preferentially only because they are large. The connections consist of two scenarios. First, the miRNA(s) in a star graph/cluster has existing connections to the mRNA(s) in the other star graph/cluster. Second, the mRNA(s) in a star graph/cluster has existing connections to the miRNA(s) in the other star graph/cluster. The mathematical meaning of the cross weight is to detect the compounded connections between every two cluster candidates. An example diagram of a cross weight calculation for two disjoint K_1,25_ (S_25_) star graphs is shown in Fig. 12.

Then the cross weight of vertices denoting clusters or star graphs in the auxiliary graph are calculated before each round of the blossom algorithm. The calculated cross weights are assembled into an edge list with vertex names of star graphs or clusters as first two column names and cross weights as the third column name. If a row of cross weight edge list has zero value cross weight, the row of the two star graphs or clusters is discarded. The edge list of non-zero cross weight is the input of each iteration of the blossom algorithm.

### The blossom algorithm for merging partitions

Taking the edge list of cross weight of 312 K_1,k_ (S_k_) star graphs as initial input, the blossom algorithm is repeatedly applied to match and merge clusters. After applying each round of the the blossom algorithm to the cross weight edge list, the maximum edge-weighted matching of vertices of clusters in the auxiliary graph is found. If there is no match for some star gaphs or clusters, those star graphs or clusters are put aside and not used in the next round of the blossom algorithm. Then, every two matched star graphs or clusters are merged to form new clusters. Cross weights of every two newly merged clusters are calculated for the next round of blossom algorithm. Then the blossom algorithm is repeatedly applied to the edge list of cross weight of vertices of clusters in newly formed auxiliary graph. The output of each round of blossom is a communities object in R programming language containing merged star graphs or clusters. The blossom algorithm was implemented using NetworkX package [31] in Python programming language.

### Evaluation of six edge weight formulas by MWMM

As for the six different edge weight formulas, it would be interesting to check how different the obtained final partitions are. If the traditional clustering algorithm is used to see the final partitions, all the edges will be added to the graph, and thereby, the final partitions of six edge weights formula would be identical. Furthermore, the final partition will have 20,661 edges in the case of BRCA such that the graph would be indistinguishable. Instead, certain number of edges/steps, say, 38 edges/steps, can be used to compare the resultant partitions of traditional hierarchical clustering algorithm. However, the partitions from six different edge weight formula might have different number of nodes. Thereby it is hard to use the global evaluation metrics such as the adjusted rand index to compare the similarity of the partitions. The adjusted Rand index (ARI) can be used to measure the similarity of the two communities of clusters. ARI needs the knowledge of the ground truth classes, which is not available in real data set or requires manual annotation such as in the supervised learning (https://scikit-learn.org/stable/modules/clustering.html). The ARI has a value close to 0.0 for random labeling independently on the number of clusters and samples and has a value exactly 1.0 when the clusters are identical (https://scikit-learn.org/stable/modules/generated/sklearn.metrics.adjusted_rand_score.html) [32]. So in this study the ARI cannot be used to tell whether the predicted clusters are similar to the true clusters. But ARI can be used to compare the similarity of resultant clusters of six weight edge formulas produced by the MWMM pipeline.

## Results

### Evaluation of the six kinds of edge weights by traditional hierarchical clustering algorithm

The traditional hierarchical clustering algorithm can subjectively cluster the miRNA-mRNA pairs by filtering the top-weighted edges in the graph. It can also be used to evaluate the proposed six edge weight formulas. We calculated the six kinds of edge weights and output the results as the edge list with miRNA node, mRNA node, and their edge weight, as is shown in Fig. 2 and Table 2. Then we ran the traditional clustering algorithm to cluster the miRNA-mRNA pairs based on six proposed edge weights.

**Fig. 13:**
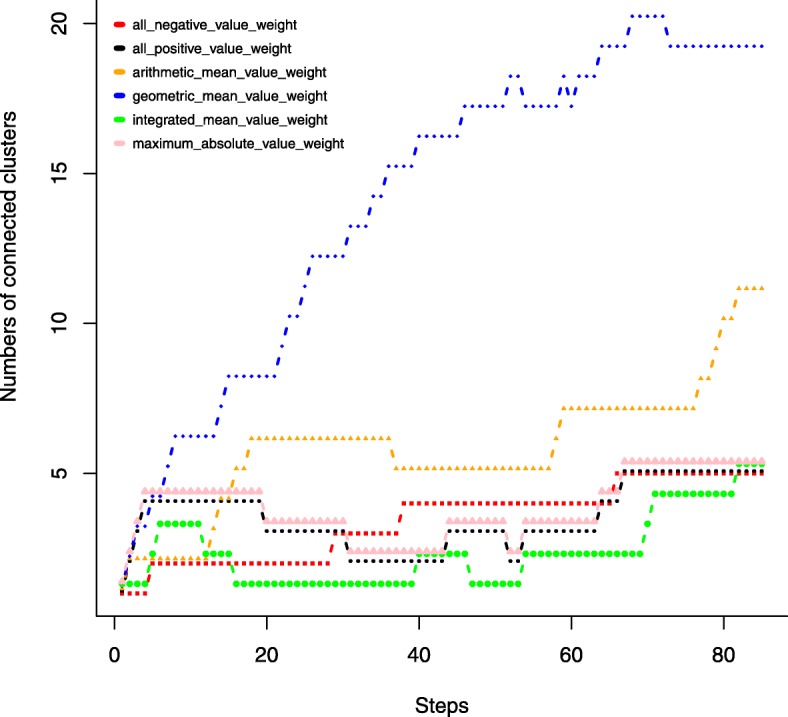
numbers of connected or disjoint clusters derived from traditional hierarchical clustering algorithm with different steps n under six edge weight formulas. The step value n is the number of the top weighted edges added to the bipartite graph

Given a specific number of steps in the traditional hierarchical clustering, smaller number of disjoint clusters suggest that more high-weighted miRNA-mRNA interactions are clustered. If the miRNA-mRNA pairs with large edge weights fall into more disjoint small clusters, there will be a larger number of disjoint clusters, which suggest that there is no coordinated interaction within the clusters. From Fig. 13, we can see that under most of the step values, the integrated mean value weight has the fewest disjoint clusters and thereby is the preferable formula in this study. Although we chose the integrated mean value weight formula, researchers facing different data can still propose other formulas. These formulas should simultaneously combine the miRNA-mRNA expressional correlation coefficient changes from in normal to in tumor.

### Traditional hierarchical clustering algorithm on top integrated mean value weight edges

**Fig. 14:**
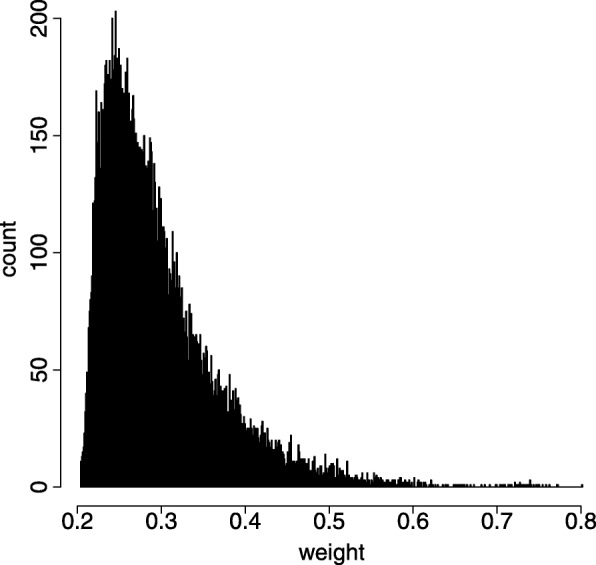
Histogram of distribution of integrated mean value weight in BRCA

**Fig. 15:**
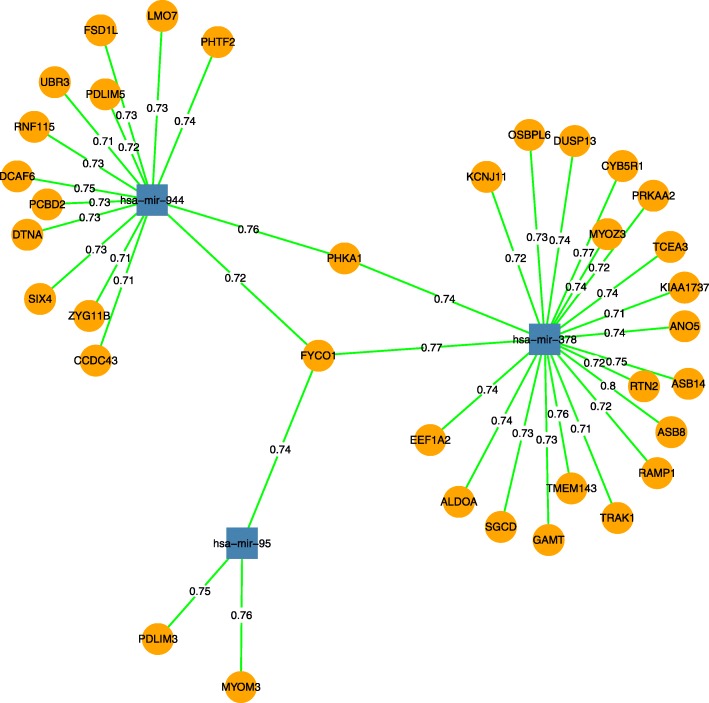
The traditional hierarchical clustering resultant sub-bipartite graphs run with 38 steps based on the integrated mean value weight. The numbers on the edges are edge weights of the integrated mean value weight. A green edge denotes the correlation coefficient change from positive in normal to negative in tumor. A red edge denotes the correlation coefficient change from negative in normal to positive in tumor. The circular vertices are mRNA and the rectangular vertices are miRNA

Since we chose integrated mean value weight to quantify the correlation change as edge weight of the miRNA-mRNA bipartite graph thereafter, we wanted to see how the clusters derived from traditional hierarchical clustering algorithm look like. Since traditional hierarchical clustering algorithm selects the top weighted edges subjectively by users’ setting, it is sensible to get a threshold of edge number. Therefore, we plot a histogram to show the distribution of integrated mean value weight in Fig. 14. From Fig. 14, we can see that there are 38 edge weights greater than 0.7, so we subjectively ran 38 steps of traditional hierarchical clustering algorithm on the miRNA-mRNA pairs with the integrated mean value weights. In other words, we selected the top 38 edge-weighted miRNA-mRNA pairs to form a new bipartite graph that is shown in Fig. 15. As a comparison, top 38 edge-weighted miRNA-mRNA pairs of all six edge weight formulas clustered by traditional hierarchical clustering algorithm are provided in Additional file 1

From Fig. 15, we can see that using the integrated mean value weight, the top 38 weighted edges are all green color, which means that in tumor all the three miRNAs inhibit the target mRNAs whereas in normal all these miRNAs are positively correlated with their target mRNAs. Larger weights are supposed to represent the bigger correlation inversion from normal to tumor. The same correlation change direction suggests that these miRNA and mRNAs are likely to be affected by the common causal factors and that the miRNAs playing suppressive roles to their target mRNAs is a characteristics of the cancer development.

### Literature spot-checks of the most enriched miRNAs

To study function of the three most enriched miRNAs based on the integrated mean weight edge shown in Fig. 15, we looked up these miRNAs in the literature. We found that all three miRNAs are functionally related to cancer [33–35]. Their functions are listed in Table 5

**Table 5:** Functions of the three most enriched miRNAs in the literature

miRNA	Function	Role	Cancer type	Reference
*miR-378*	suppresses the proliferation, migration and invasion	tumor suppressor	colon cancer	[33]
*miR-944*	promotes cell proliferation and tumor metastasis	oncogenic	breast cancer	[34]
*miR-95*	inhibit tumor cell apoptosis and increase cellular proliferation	oncogenic	non-small cell lung cancer	[35]

### Apply MWMM pipeline in BRCA

The whole MWMM pipeline first calculates edge weights from table of edge list of miRNA-mRNA pairs with their expression T_CC and N_CC, exemplified in Table 1. The calculated edge weights of integrated mean value weight are exemplified in Table 2. The raw edge list contains 20,661 pairs of miRNA and mRNAs, including 312 unique miRNAs and 7874 unique mRNAs. This raw edge list is converted to raw bipartite graph matrix for the Hungarian algorithm to run on. The number of Hungarian algorithm iterations is 202 rounds. The merging process yields 312 K_1,k_ (S_k_) star graphs, from which the blossom algorithm is repeatedly applied to match and merge clusters. The blossom algorithm run 8 times to eventually merge 312 starting star graphs or clusters into a single cluster. One single cluster doesn’t make sense for the purpose of clustering, but the clusters of last iteration of Hungarian algorithm and each round of blossom algorithm are output to communities objects in R programming language. Users can use mathematical and biological metrics to select clusters derived from Hungarian algorithm or from first several rounds of blossom algorithm to achieve the trade-off between cluster size and cluster number.

### Evaluation of six edge weight formulas by MWMM

To see the effects of six different edge weight formulas, ARI was used to compare the similarity of resultant clusters based on six weight edge formulas produced by MWMM pipeline. MWMM started with six different edge weight formulas and produced six communities structures of clusters, respectively. Communities is a structure in igraph package in R programming language to represent clusters. We compared similarity of two communities structures of clusters derived from every two different edge weight formulas using ARI. The communities structures of clusters produced by Hungarian algorithm and blossom 01 of the MWMM approach were shown as examples in Tables 6 and 7, respectively, to tell whether different edge weight formulas lead to different communities structures. From Tables 6 and 7, we found that the communities structure of clusters derived from every two edge weight formulas using Hungarian or blossom algorithm 01 were very similar, because their ARI values are in the same order of magnitude The overall similarity using blossom 01 is higher than that using Hungarian algorithm, perhaps because the blossom algorithm merge the clusters generated from Hungarian algorithm, and thereby the communities structures of clusters are more similar using blossom algorithm than these using Hungarian algorithm. Since ARI only compares clusters based on their topological structures, ignoring their edge weights, ARI is not a suitable metrics to select a good edge weight formula, whereas traditional hierarchical clustering algorithm did the job.

**Table 6:** Similarities of communities structures of clusters derived from six edge weight formulas using Hungarian algorithm in the MWMM pipeline. All combinations of every two edge weight formulas are listed. Their similarity score of ARI are calculated

from	to	ARI
all_negative_value_weight	all_positive_value_weight	0.025
all_negative_value_weight	arithmetic_mean_value_weight	0.022
all_negative_value_weight	geometric_mean_value_weight	0.027
all_negative_value_weight	integrated_mean_value_weight	0.027
all_negative_value_weight	maximum_absolute_value_weight	0.026
all_positive_value_weight	arithmetic_mean_value_weight	0.03
all_positive_value_weight	geometric_mean_value_weight	0.037
all_positive_value_weight	integrated_mean_value_weight	0.036
all_positive_value_weight	maximum_absolute_value_weight	0.039
arithmetic_mean_value_weight	geometric_mean_value_weight	0.04
arithmetic_mean_value_weight	integrated_mean_value_weight	0.062
arithmetic_mean_value_weight	maximum_absolute_value_weight	0.046
geometric_mean_value_weight	integrated_mean_value_weight	0.03
geometric_mean_value_weight	maximum_absolute_value_weight	0.036
integrated_mean_value_weight	maximum_absolute_value_weight	0.088

**Table 7:** Similarities of communities structures of clusters derived from six edge weight formulas using blossom 01 in the MWMM pipeline. All combinations of every two edge weight formulas are listed. Their similarity score of ARI are calculated

from	to	ARI
all_negative_value_weight	all_positive_value_weight	0.526
all_negative_value_weight	arithmetic_mean_value_weight	0.527
all_negative_value_weight	geometric_mean_value_weight	0.551
all_negative_value_weight	integrated_mean_value_weight	0.557
all_negative_value_weight	maximum_absolute_value_weight	0.531
all_positive_value_weight	arithmetic_mean_value_weight	0.541
all_positive_value_weight	geometric_mean_value_weight	0.565
all_positive_value_weight	integrated_mean_value_weight	0.511
all_positive_value_weight	maximum_absolute_value_weight	0.58
arithmetic_mean_value_weight	geometric_mean_value_weight	0.542
arithmetic_mean_value_weight	integrated_mean_value_weight	0.5
arithmetic_mean_value_weight	maximum_absolute_value_weight	0.541
geometric_mean_value_weight	integrated_mean_value_weight	0.58
geometric_mean_value_weight	maximum_absolute_value_weight	0.545
integrated_mean_value_weight	maximum_absolute_value_weight	0.541

### Mathematical metrics of MWMM-derived clusters

**Fig. 16:**
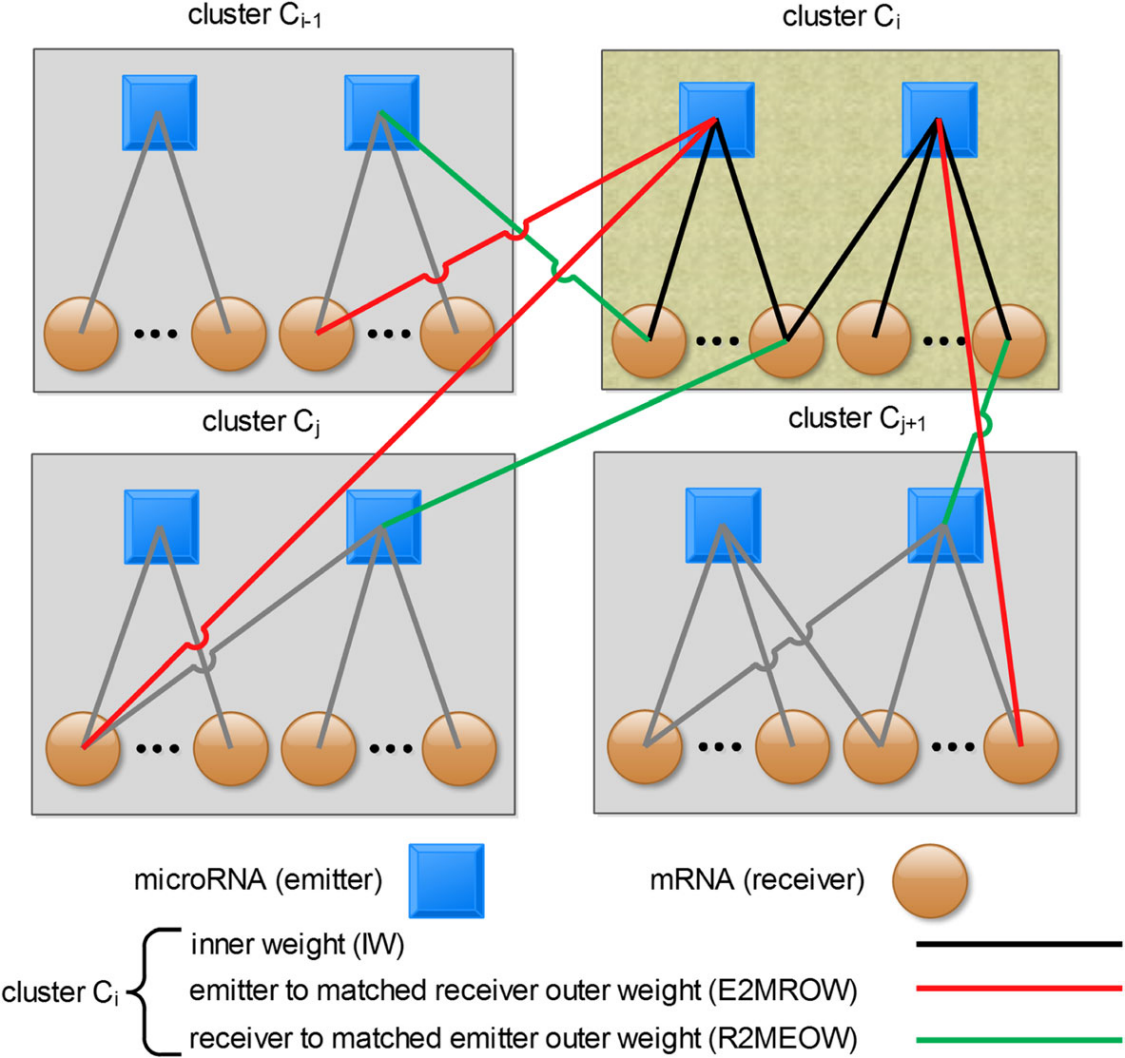
The diagram of inner weight and outer weight of a given cluster C_i_. The outer weights are classified into two categories: emitter to matched receiver outer weight and receiver to matched emitter outer weight

A well-partitioned cluster should have more weighted connections inside the cluster and fewer weighted connections to any other outside clusters, so that the members of the cluster are more similar or linked to each other than to the members of other outside clusters. This characteristics of clusters leads to the clustering metrics used in this study. We define three weight notations to describe the connections between the microRNA (emitter) and mRNA (receiver) within and across clusters to measure the inner weight and outer weight of clusters generated by the Hungarian algorithm and the blossom algorithm. The three definitions are listed in Table 8. The diagram of the calculation of inner and outer weights are portrayed in Fig. 16.

**Table 8:** definition of three different weights for a given cluster candidate C_i_

Notation	Abbreviation	the sum of edge-weight connections
inner weight	IW	between miRNAs and mRNAs within a cluster candidate C_i_
emitter to matched receiver outer weight	E2MROW	from miRNAs in the cluster candidate C_i_ to mRNAs in all other cluster candidates C_j_ where j ≠ i
receiver to matched emitter outer weight	R2MEOW	from mRNAs in the cluster candidate C_i_ to miRNAs in all other cluster candidates C_j_ where j ≠ i

Accordingly, we propose two conditions to validate the mathematical significance of a candidate cluster C_i_. Condition one specifies IW > E2MROW. Condition two specifies 2 × IW > E2MROW + R2MEOW. It is noteworthy that in the condition two the inner weight should be doubled, because the condition two’s outer weights measure the connections from miRNAs inside a cluster to mRNAs outside that cluster as well as the mRNAs inside that cluster to the miRNAs outside that cluster; correspondingly, the inner weight of a cluster should also be measured twice to characterize the connections from miRNAs inside a cluster to mRNAs inside that cluster as well as from mRNAs inside that cluster to miRNAs inside that cluster. The difference of meeting condition one and condition two may result from that condition one is based on only the miRNA side, whereas condition two is based on both miRNA and mRNA sides. Condition one is less stringent than condition two, because condition one only compares the inner and the outer connections to the miRNAs inside a cluster, whereas condition two compares the inner and the outer connections to both miRNAs and mRNAs inside a cluster. Therefore, condition one is easily met by fewer rounds of merging algorithm.

**Fig. 17:**
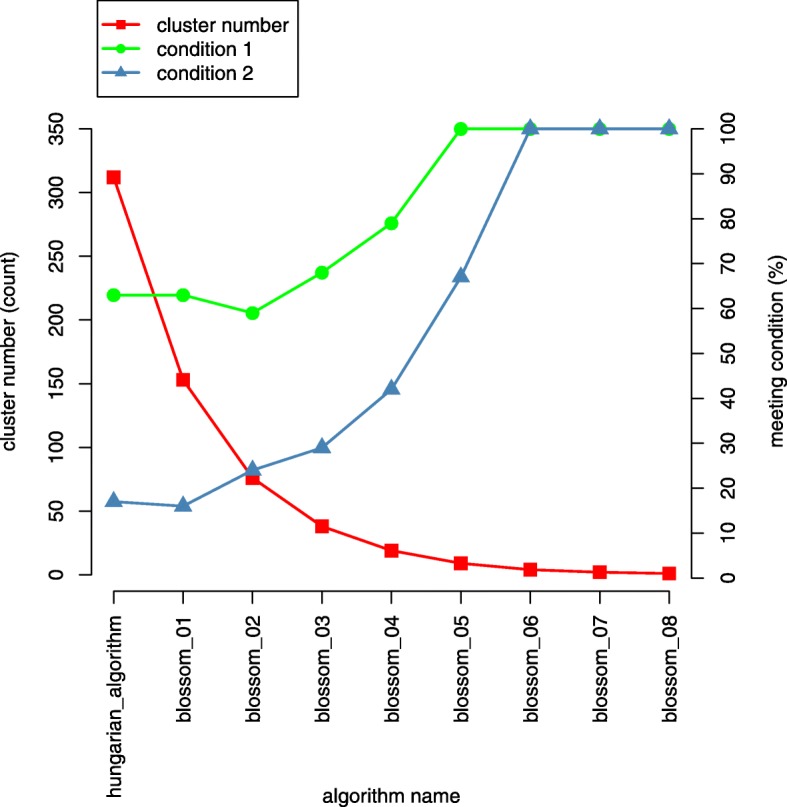
The change of cluster numbers and condition satisfactions as more merging rounds are applied. The MWMM approach is applied to BRCA data set

The whole MWMM procedure consists of continuous iterations of the Hungarian algorithm and several rounds of the Blossom algorithm. The MWMM procedure tries to merge existing clusters to generate new clusters that have greater inner weight than the outer weight, seen in Fig. 17. If we keep merging, eventually there will be one or several very dense clusters, which have small outer weight. Thereby, we need to make a trade–off between the sizes and density of the clusters. In other words, we want denser clusters with proper sizes.

From Fig. 17, we can see that continuous application of the Hungarian algorithm produces 312 star graphs. Afterwards, using eight rounds of application of Blossom algorithm. The 312 star graphs/clusters are merged to one cluster round by round. The merger effects are evaluated by the above-mentioned condition one and condition two. We can see that as more rounds of merging algorithm are applied, the number of clusters first decreases dramatically and then tends to be stable; the clusters of different sizes satisfying condition one and/or condition two are produced by the MWMM procedures and the percent of clusters that meets the condition one (IW > E2MROW) and condition two (2 × IW > E2MROW + R2MEOW) gradually increases to 100% and becomes stable. The detailed metrics of the final clusters defined in Table 8 are provided in Additional file 2.

### Kyoto encyclopedia of genes and genomes (KEGG) analysis of the clustering results

**Fig. 18:**
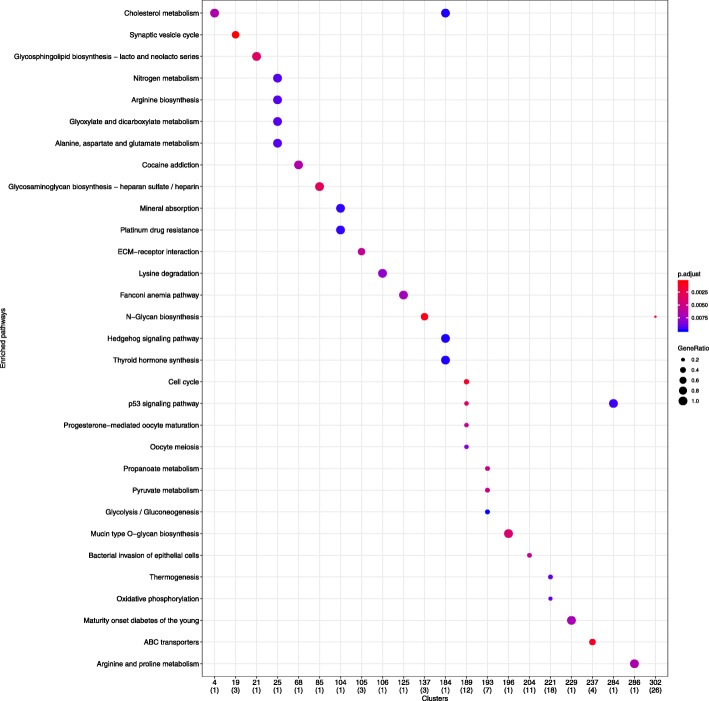
KEGG pathway enrichment of clusters derived from the Hungarian algorithm result in BRCA. The upper row of the x axis label is the ordinal number of the 312 clusters that are significant enriched KEGG pathways. The lower row of the x axis label is value of n in geneRatio that is defined as k/n, where n is the size of the list of genes of interest and k is the number of genes within that list which are annotated to the node. Technical details of geneRatio refers to instructions of DOSE packages [37]

**Fig. 19:**
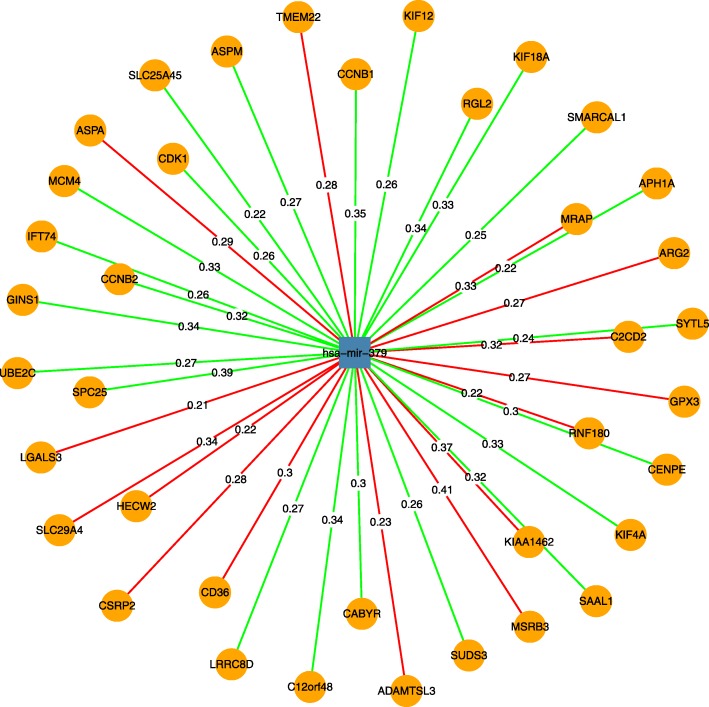
The miRNA and mRNA members of 189th cluster (star graph) derived from the Hungrian algorithm. The numbers on the edges are edge weights of the integrated mean value weight. A green edge denotes the correlation coefficient change from positive in normal to negative in tumor. A red edge denotes the correlation coefficient change from negative in normal to positive in tumor. The circular vertices are mRNA and the rectangular vertices are miRNA

KEGG function analysis shows the biological significance of genes that are potentially regulated by miRNAs in the derived clusters. The biological factors enriched in the clusters provide a new viewpoint on how mRNA–miRNA pairs contribute to cancers. Functional analysis of genes in clusters is implemented using clusterProfiler, an R package for comparing biological themes among gene clusters [36]. For example, in Fig. 18, we analyze 312 clusters derived from the Hungarian algorithm result and get 22 clusters enriched in KEGG pathways with pvalueCutoff = 0.01 and qvalueCutoff = 0.05. For example, genes in 189th cluster are enriched in cell cycle, p53 signaling pathway, progesterone−mediated oocyte maturation, and oocyte meiosis, suggesting the theme of the genes in the cluster related to cancer. The members of 189th cluster (star graph) are visualized in Fig. 19, where the internal node miRNA hsa-mir-379 is reported to be a tumor suppressor playing a role in inhibiting cell proliferation, migration, and invasion in breast cancer [38], cervical cancer [39], glioma [40], non-small cell lung cancer [41], bladder cancer [42], osteosarcoma [43], hepatocellular carcinoma [44], gastric cancer [45]. The genes interacting with hsa-mir-379 in the cluster is worth further experimental exploration, for example, *CCNB1, MCM4, CCNB2,* and *CDK1* that are involved in cell cycle.

### Clustering method comparison

To see if our MWMM approach surpasses existing clustering methods, we need to conduct performance comparisons. Which clustering approaches are suitable for comparison? First, the MWMM method is a downstream analysis approach taking certain input format, an edge list with miRNA vertex name, mRNA vertex name, and their edge weight. The integrated mean value weight characterizes the correlation change in two conditions. Therefore, the clustering approaches that only consider one condition like MAGIA2 or miRMAP are not comparable to the MWMM. Second, other known clustering algorithm might not fit the data structure of bipartite graph in a form of edge list. For example, in a study using time course mRNA microarray data, a non-linear primary component analysis (PCA) neural network was used to extract the feature vector that was afterwards fed into a probabilistic principal surfaces (PPS) model to find and visualize latent variables or clusters of genes that were afterwards merged by an agglomerative clustering algorithm based on negentropy information. This negentropy clustering (NEC) algorithm can automatically determine the cluster of numbers [46], so it is better than the traditional hierarchical clustering algorithm that needs subjective determination of the cluster number. However, this study concentrates in the miRNA-mRNA interactions, in which a bipartite graph is constructed, so the clustering approaches like PPS-NEC [46], k-means [47], or WGCNA [48] that have been used to find gene expression “modules” or clusters are unsuitable for comparison. Third, the miRNA-mRNA interaction bipartite graph is not a connected graph, and thereby, some clustering algorithms like minimum spanning tree cannot be applied. Considering the above-mentioned constraints, we choose louvain, fast_greedy, walktrap, leading_eigen, label_propagation, and edge_betweenness to compare with the MWMM approach. Implementations of these clustering approaches are derived from igraph package in R programming language.

The biological validation would benefit from a systematic methodology in addition to literature spot-checks. Thereby, we biologically validate the derived clusters by calculating their average Gene Ontology (GO) term similarity distance scores. The GO similarity scores would give an idea of how the genes within a cluster or across clusters are functionally related or similar. Based on the definition of clustering, elements within a cluster are more similar or linked than the elements among clusters in some traits, for example, GO term similarity. Thereby, clusters identified by a good clustering algorithm should have higher intra-cluster GO similarity distance scores and lower inter-cluster GO similarity distance scores. In other words, the difference between intra-cluster GO similarity score and inter-cluster GO similarity score should be higher for a good clustering algorithm.

**Fig. 20:**
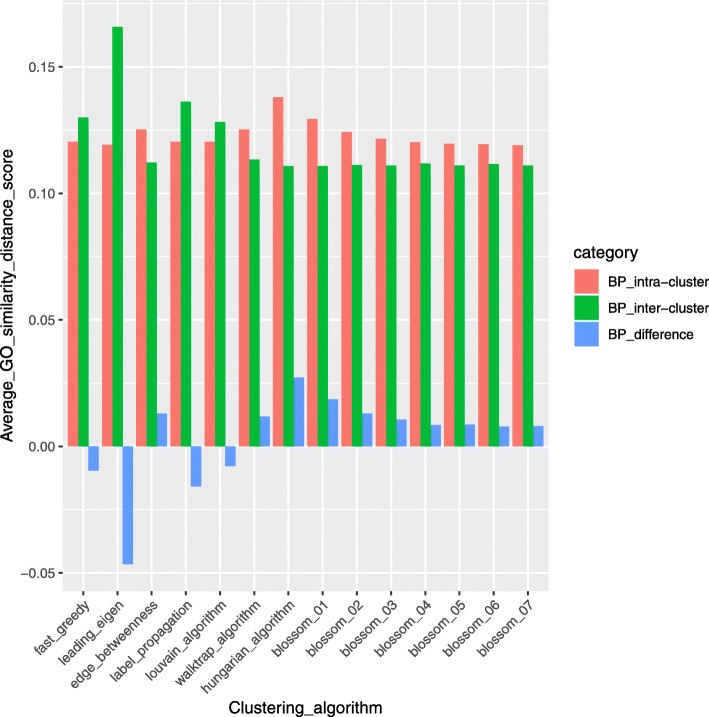
Average GO term (Biological Process) similarity distance scores of different algorithms in BRCA. In the legend, intra-cluster means GO similarity scores within a cluster; inter-cluster means GO similarity scores across clusters; difference is the difference between intra-cluster and inter-cluster GO similarity scores

**Fig. 21:**
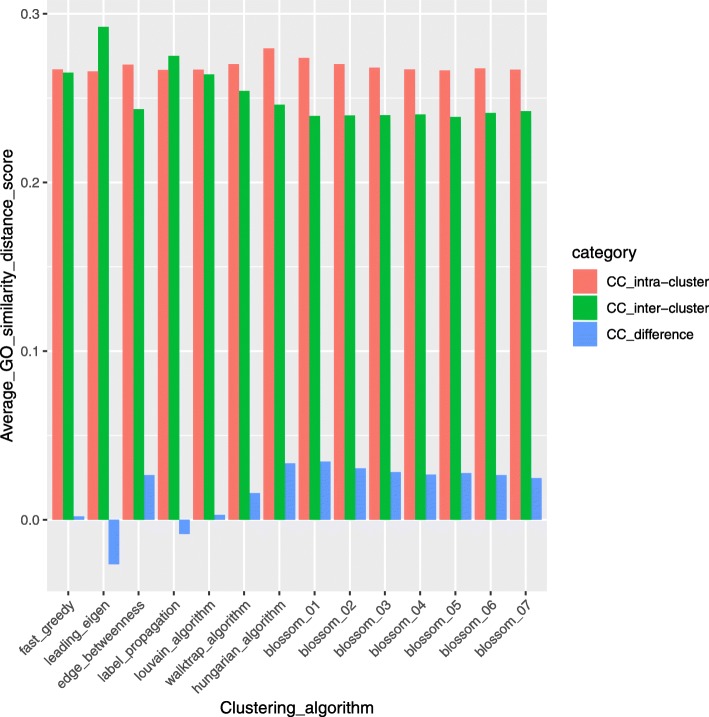
Average GO term (Cellular Component) similarity distance scores of different algorithms in BRCA. In the legend, intra-cluster means GO similarity scores within a cluster; inter-cluster means GO similarity scores across clusters; difference is the difference between intra-cluster and inter-cluster GO similarity scores

**Fig. 22:**
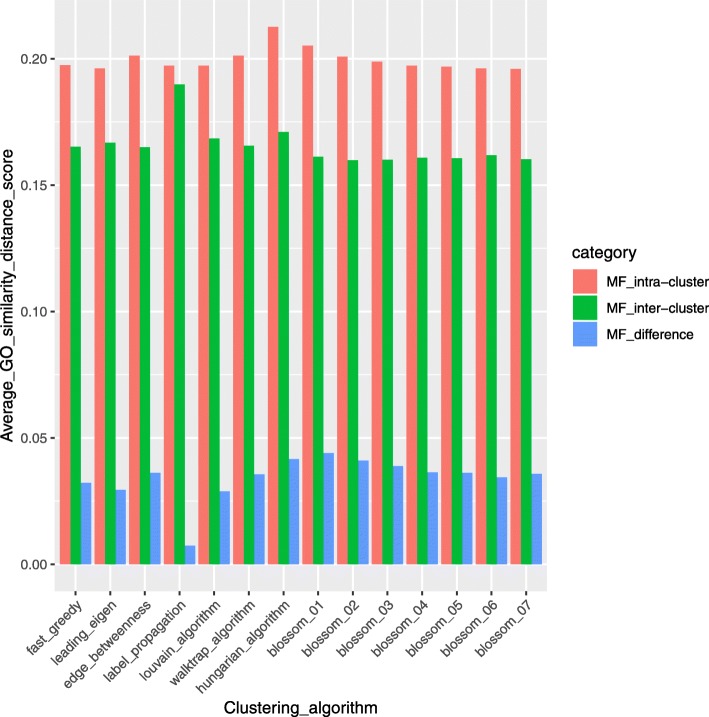
Average GO term (Molecular Function) similarity distance scores of different algorithms in BRCA. In the legend, intra-cluster means GO similarity scores within a cluster; inter-cluster means GO similarity scores across clusters; difference is the difference between intra-cluster and inter-cluster GO similarity scores

To compare and evaluate clusters generated by different clustering algorithms in BRCA, the GO similarity distance scores of genes in the clusters are calculated using GOSemSim, an R package for measuring semantic similarity among GO terms and gene products [49]. GO similarity distance score is calculated in three categories of GO terms: molecular function (MF) describing molecular activities of gene products, cellular component (CC) describing where gene products are active, and biological process (BP) describing pathways and larger processes made up of the activities of multiple gene products. From Figs. 20, 21, and 22, we can see that compared to other algorithms, the Hungarians or Blossom algorithm 01 have relatively higher intra-cluster similarity and relatively lower inter-cluster similarity in all three GO term categories. This result shows the advantage of MWMM approach over other approaches in biological meaning.

Besides comparison with different methods like Louvain, fast_greedy, walktrap, leading_eigen, label_propagation, and edge_betweenness algorithms in terms of GO terms, a mathematical comparison in term of strength of the connection inside the cluster and outside the clusters is also meaningful. So we calculated the inner weight and outer weight and conditions defined in Table 8 for the MWMM pipeline and other compared algorithms. Different algorithms produced different number of clusters in the resultant communities structures. To summarize, the IW, E2MROW, and R2MEOW of clusters produced using each algorithm were averaged, and the percent of how many clusters produced using each algorithm meet condition 01 or 02 were also calculated, respectively. The result summary is listed in Table 9. From Table 9, we can see that louvain, fast_greedy, and leading_eigen algorithms yielded clusters with the larger inner weights relative to outer weights and high percent of condition 01 and 02 satisfaction. By comparison, the Hungarian and blossom algorithms in the MWMM approach at the beginning did not produce clusters with the larger inner weights relative to outer weights and high percent of condition 01 and 02 satisfaction, however, as the merger process went on in the MWMM, the Hungarian and blossom algorithms in the MWMM approach gradually generated clusters with the larger inner weights relative to outer weights and high percent of condition 01 and 02 satisfaction. These phenomena comply with expectations, because all the clustering algorithms try to make clusters based on mathematical criteria, while clusters are defined as inner connections or similarities greater than the outer connections or similarities.

**Table 9:** Average inner weight and outer weights of clusters produced using each algorithm and how many percent of clusters meet the conditions

	Average IW	Average E2MROW	Average R2MEOW	Condition 01 true percent	Condition 02 true percent
hungarian_algorithm	0.243	0.249	0.589	63.14%	16.99%
blossom_01	0.282	0.296	0.552	62.75%	15.69%
blossom_02	0.305	0.303	0.513	59.21%	23.68%
blossom_03	0.331	0.295	0.477	68.42%	28.95%
blossom_04	0.366	0.288	0.42	78.95%	42.11%
blossom_05	0.42	0.268	0.346	100.00%	66.67%
blossom_06	0.508	0.193	0.223	100.00%	100.00%
blossom_07	0.598	0.126	0.127	100.00%	100.00%
blossom_08	0.725	0	0	100.00%	100.00%
fast_greedy	0.289	0.14	0.141	86.36%	95.45%
leading_eigen	0.27	0.165	0.111	90.00%	90.00%
edge_betweenness	0.266	0.331	0.3	46.32%	37.89%
label_propagation	0.243	0.162	0.069	80.00%	100.00%
louvain_algorithm	0.302	0.157	0.123	95.24%	100.00%
walktrap_algorithm	0.228	0.173	0.164	70.80%	81.42%

The running speed of different algorithms were compared by running on the same data set: the edge list of miRNA and mRNAs with integrated mean value edge weight from BRCA. The running time was recorded respectively and listed in Table 10. From Table 10, we can see that the label-propagation, Louvain, fast_greedy, leading_eigen, and walkstrap algorithms are fast. Our hungagrian_blossom (MWMM) approach is acceptable. Edge_betweenness algorithm is slow.

**Table 10:** Running time of different algorithms on BRCA data set edge list of miRNA and mRNAs with integrated mean value edge weight

Algorithm name	Running time (second)
label-propagation	0.048
louvain_algorithm	0.062
fast_greedy	0.657
leading_eigen	1.073
walkstrap	3.078
hungagrian_blossom (MWMM)	449.232
edge_betweenness	125,278

### Clustering algorithm validation on test data sets

The MWMM approach is developed using BRCA as training data set. Can this approach also applied to some test data sets and achieve similar clustering results in terms of mathematical cluster traits and biological meaning? To answer this question, we ran MWMM approach and other six foregoing clustering algorithms on other 14 cancer types: Bladder Urothelial Carcinoma (BLCA), Colon adenocarcinoma (COAD), Esophageal carcinoma (ESCA), Head and Neck squamous cell carcinoma (HNSC), Kidney Chromophobe (KICH), Kidney renal clear cell carcinoma (KIRC), Kidney renal papillary cell carcinoma (KIRP), Liver hepatocellular carcinoma (LIHC), Lung adenocarcinoma (LUAD), Lung squamous cell carcinoma (LUSC), Prostate adenocarcinoma (PRAD), Stomach adenocarcinoma (STAD), Thyroid carcinoma (THCA), and Uterine Corpus Endometrial Carcinoma (UCEC). Similar to BRCA, the input table of the 14 cancer types exemplified in Table 1 were derived from results of our previous study [18].

**Fig. 23:**
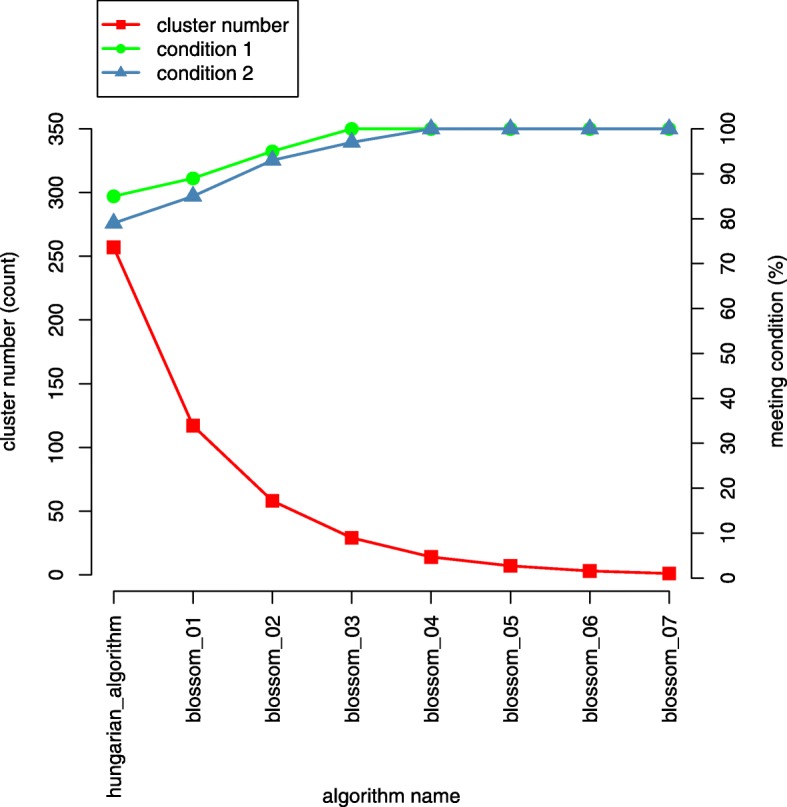
the change of cluster numbers and condition satisfactions as more merging rounds are applied. The MWMM approach is applied to KIRP data set

We find that similar to in BRCA the MWMM can also detect clusters that has internal weights greater than or equal to external weights in the test data sets of 14 cancer types. Graph of inner weights, outer weights, and cluster sizes of KIRP is drawn in Fig. 23 as an example. Graphs of other 13 cancer types are supplied in Additional file 3.

We also tried to find out whether the MWMM approach can cluster miRNAs and mRNAs such that the difference between intra-cluster and inter-cluster average GO similarity distance score is relatively larger compared to other algorithm results. The clustering algorithms that obtain the highest differences between intra-cluster and inter-cluster average GO similarity distance score in each GO term category in each cancer type are summarized in Table 11. We can see that in MWMM has the best GO metrics in terms of BP in 11 out of 15 cancer types, CC in 13 out of cancer types, and MF in 14 out of 15 cancer types. The results suggest that the MWMM are also effective in other cancer types, though it is not always the best. The supporting materials for Table 11 are provided in Additional file 4.

**Table 11:** summary of which clustering approaches achieve the highest difference of intra-cluster and inter-cluster average GO similarity distance score in three GO term categories in 15 cancer types

cancer_type	top_BP_difference	top_CC_difference	top_MF_difference
BLCA	MWMM	MWMM	MWMM
BRCA	MWMM	MWMM	MWMM
COAD	MWMM	edge_betweenness	leading_eigen
ESCA	label_propagation	MWMM	MWMM
HNSC	MWMM	MWMM	MWMM
KICH	MWMM	MWMM	MWMM
KIRC	MWMM	MWMM	MWMM
KIRP	MWMM	MWMM	MWMM
LIHC	MWMM	MWMM	MWMM
LUAD	MWMM	MWMM	MWMM
LUSC	label_propagation	MWMM	MWMM
PRAD	MWMM	leading_eigen	MWMM
STAD	MWMM	MWMM	MWMM
THCA	label_propagation	MWMM	MWMM
UCEC	label_propagation	MWMM	MWMM

## Discussion

There are some miRNA-mRNA clustering studies, however, these studies did not focus on the expression correlation coefficient changes of miRNA-mRNA pairs that are inverse from in normal to in tumor. The miRNAs and mRNAs can be clustered based on their expression correlation coefficient changes under the assumption that the changes are not random but caused by factors involved in cancer development. Hence, we tried to capture and cluster these miRNA-mRNA interactions.

To simultaneously quantify the changes, we proposed integrated mean value weight that increases the contrast of values in the data as well as other five edge weight formula as comparison or control. Then the subjective traditional hierarchical clustering algorithm was used to evaluate the advantages of different edge weight formulas. After evaluation, integrated mean value weight was favored because it can produce more connected clusters at certain steps. We did not just use the traditional hierarchical clustering algorithm only to cluster miRNA and mRNA pairs in this study; instead, we only use it as a tool to evaluate the edge weight formulas. This is because traditional hierarchical clustering algorithm is subjective and thereby makes the researchers feel difficult to determine the cluster number. Furthermore, traditional hierarchical clustering algorithm only cluster the top miRNA-mRNA pairs, and thereby it doesn’t reach a global optimal clustering that should also involve the low edge weight miRNA-mRNA pairs. To get around these limitations, we proposed the maximum weighted merger method (MWMM) pipeline.

The MWMM pipeline includes continuous iterations of Hungarian algorithm and several rounds of blossom algorithm. MWMM pipeline passively clusters miRNA-mRNA pairs using maximum weighted edge matching in the bipartite graph and general graph. Based on the GO similarity results, the Hungarian algorithm or blossom 01 can produce clusters that have a good trade-off between the cluster size and GO similarity, compared to the other algorithms that produce several huge-sized clusters along with some small-sized clusters. Functional enrichment analysis such as KEGG pathway and GO terms was performed to find out the underlying factors or themes from genes in each derived cluster. For example, genes involved in p53 signaling pathway and cell cycle pathways were successfully identified.

The effectiveness of MWMM was validated both mathematically and biologically. Mathematically, the MWMM-derived clusters were analyzed with respect to their inner weights and outer weight. The percent of clusters that meet the condition one and two gradually increases as the MWMM merger process goes on. Eventually, all MWMM-derived clusters have inner weights greater than their outer weight, namely, greater inside connection than outside connection. Biologically, MWMM-derived clusters have intra-cluster’s average GO term similarity distance scores much larger than the inter-cluster’s, compared to other six algorithms. MWMM approach was also applied to other 14 cancer types and it can merge initial clusters to yield clusters that mostly keep the inner weights larger than or equal to the outer weight in other 14 cancer types. Biologically, the MWMM approach yields clusters that has relatively higher intra-cluster and relatively lower inter-cluster average GO term similarity distance scores compared to other six clustering algorithms in most of cancer types that are tested. This shows that the MWMM can also be applied to data sets other than BRCA.

In the future, more information could be integrated into MWMM pipeline. First, the expression fold change of miRNAs and mRNAs could also be considered into the edge weights of the miRNA-mRNA interactions to see the relationship between the expression fold change and correlation coefficient change. Second, the current study is configured such that it only considers the inverse correlation coefficient change, namely from positive to negative or from negative to positive. It would be interesting to see whether from high positive to low positive or from high negative to low negative matters. Third, more filters could be applied to the clustering algorithm such as filtering out the smallest weight edges of miRNA-mRNA pairs. Fourth, more underlying factors or themes of each derived clusters would be easier to be unraveled by considering other factors like gene mutations, transcription factors, long noncoding RNAs, other regulatory elements, etc. This needs incorporating literature studies and other formats of omics data.

## Conclusions

In this study, the expression correlation coefficient changes of miRNA-mRNA pairs that are inverse from in normal to in tumor were quantified by integrated mean value weight out of proposed six edge weight formulas. The integrated mean value weight was favored based on the evaluation of the subjective traditional hierarchical clustering algorithm. Then, a maximum weighted merger method (MWMM) approach combining the Hungarian algorithm and blossom algorithm was used to passively cluster the miRNA-mRNA pairs using the maximum weighted edge matching in the bipartite graph and general graph. The resultant clusters can effectively capture and enrich cancer-associated miRNA-mRNA pair candidates in different cancer types and achieve more biologically significant clusters than other existing, available algorithms such as Louvain, fast greedy, walktrap, leading eigen, label propagation, and edge betweenness algorithms. In the future study, it is worthwhile to investigate how to use the clustered miRNAs and mRNAs as candidate biomarkers for different cancer types, identify cancer driver genes, provide clues for targets of precision medicine in cancer, and develop new treatment strategies.

## Additional files

Additional files 1: Description of data: top 38 edge-weighted miRNA-mRNA pairs of all six edge weight formulas clustered by traditional hierarchical clustering algorithm are shown in the graphs. (ZIP 39 kb)

Additional files 2: Description of data: inner weight, emitter to matched receiver outer weight, receiver to matched emitter outer weight, condition 01, and condition 02 of each cluster derived from a specific algorithm in the MWMM approach. (ZIP 18 kb)

Additional files 3: Description of data: the graphs describe the change of cluster numbers and mathematical condition satisfactions as more merging rounds are applied to different cancer types by MWMM approach. The supplementary graphs have the same setting as Figs. 17 and 23 in the context. (ZIP 62 kb)

Additional files 4: Description of data: average GO similarity distance scores of intra-cluster, inter-cluster, and difference between intra-cluster and inter-cluster in each algorithm in each cancer type. (ZIP 8 kb)

## Data Availability

The source codes supporting the conclusions of this article are available in the GitHub at https://github.com/BaiLab/MWMM.

## Abbreviations

ARI: Adjusted Rand Index
BLCA: Bladder Urothelial Carcinoma
BRCA: Breast invasive carcinoma
COAD: Colon adenocarcinoma
E2MROW: Emitter to matched receiver outer weight
ENCODE: Encyclopedia of DNA Elements
ESCA: Esophageal carcinoma
GO: Gene ontology
HNSC: Head and Neck squamous cell carcinoma
ICGC: International Cancer Genome Consortium
IW: Inner weight
KEGG: Kyoto Encyclopedia of Genes and Genomes
KICH: Kidney Chromophobe
KIRC: Kidney renal clear cell carcinoma
KIRP: Kidney renal papillary cell carcinoma
LIHC: Liver hepatocellular carcinoma
LUAD: Lung adenocarcinoma
LUSC: Lung squamous cell carcinoma
MWMM: Maximum weighted merger method
N_CC: The miRNA-mRNA expressional correlation coefficients in normal.
NEC: Negentropy clustering
NGS: Next generation sequencing
PPS: Probabilistic principal surfaces
PRAD: Prostate adenocarcinoma
R2MEOW: Receiver to matched emitter outer weight
STAD: Stomach adenocarcinoma
T_CC: The miRNA-mRNA expressional correlation coefficients in tumor
TCGA: The Cancer Genome Atlas
THCA: Thyroid carcinoma
UCEC: Uterine Corpus Endometrial Carcinoma
