# Supplementary figures and images for: Clustering analysis of microRNA and mRNA expression data from TCGA using maximum edge-weighted matching algorithms
Source: BMC Med Genomics. 2019 Aug 5;12:117. doi: 10.1186/s12920-019-0562-z (PMC6683425; doi:10.1186/s12920-019-0562-z)

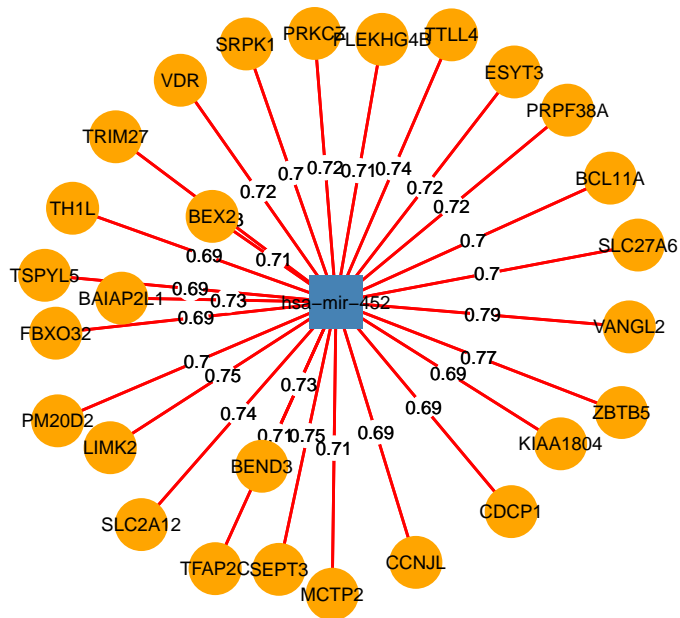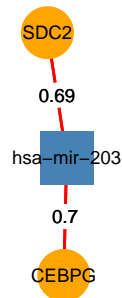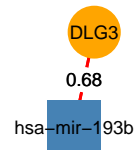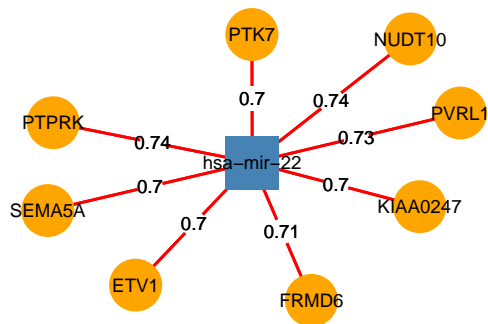

Supplement: Supplementary file 1 — Description of data: top 38 edge-weighted miRNA-mRNA pairs of all six edge weight formulas clustered by traditional hierarchical clustering algorithm are shown in the graphs. (ZIP 39 kb) [file 12920_2019_562_MOESM1_ESM.zip › all_negative_value_weight.top_38_edges.BRCAR2.pdf]

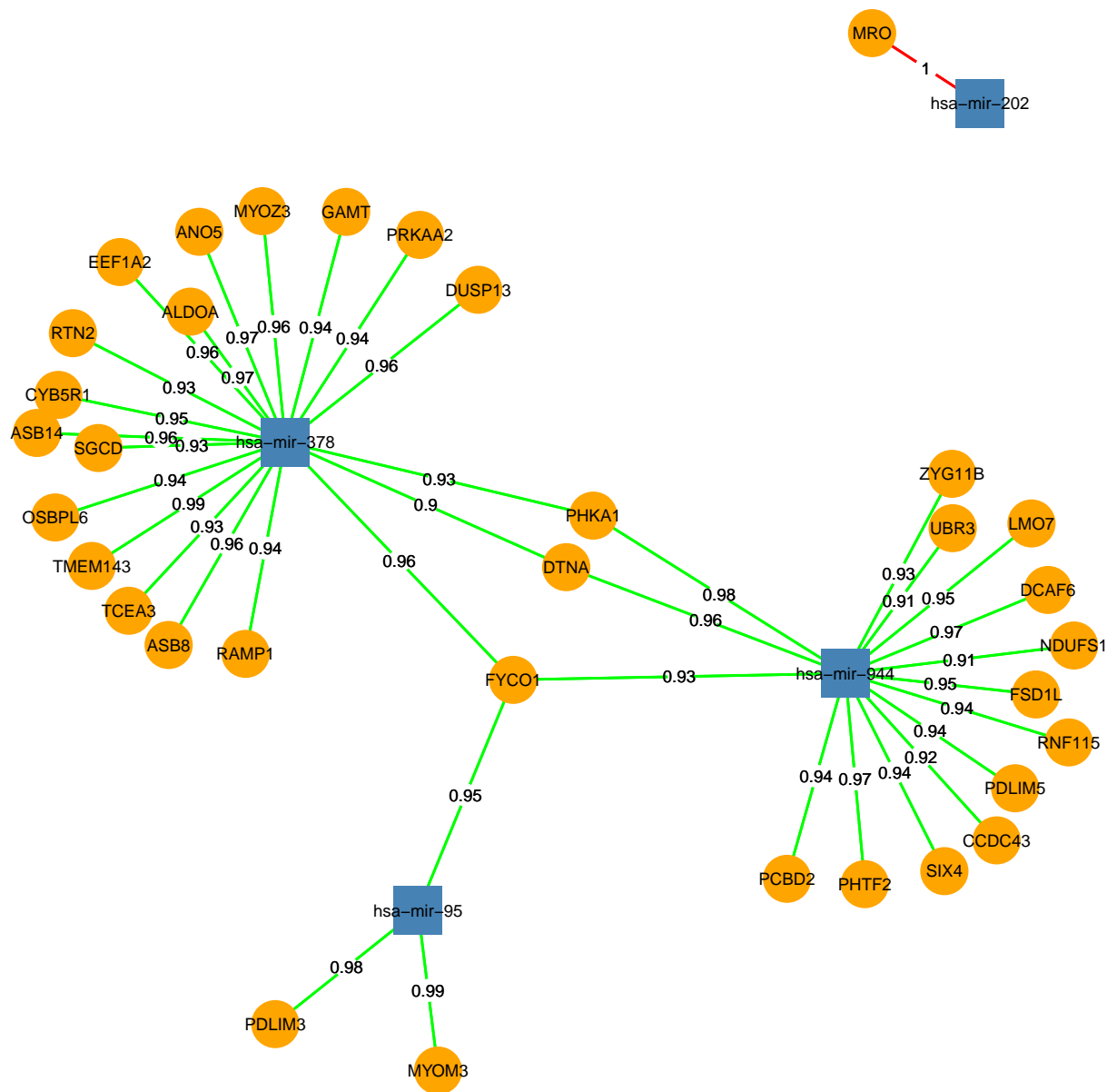

Supplement: Supplementary file 1 — Description of data: top 38 edge-weighted miRNA-mRNA pairs of all six edge weight formulas clustered by traditional hierarchical clustering algorithm are shown in the graphs. (ZIP 39 kb) [file 12920_2019_562_MOESM1_ESM.zip › all_positive_value_weight.top_38_edges.BRCAR2.pdf]

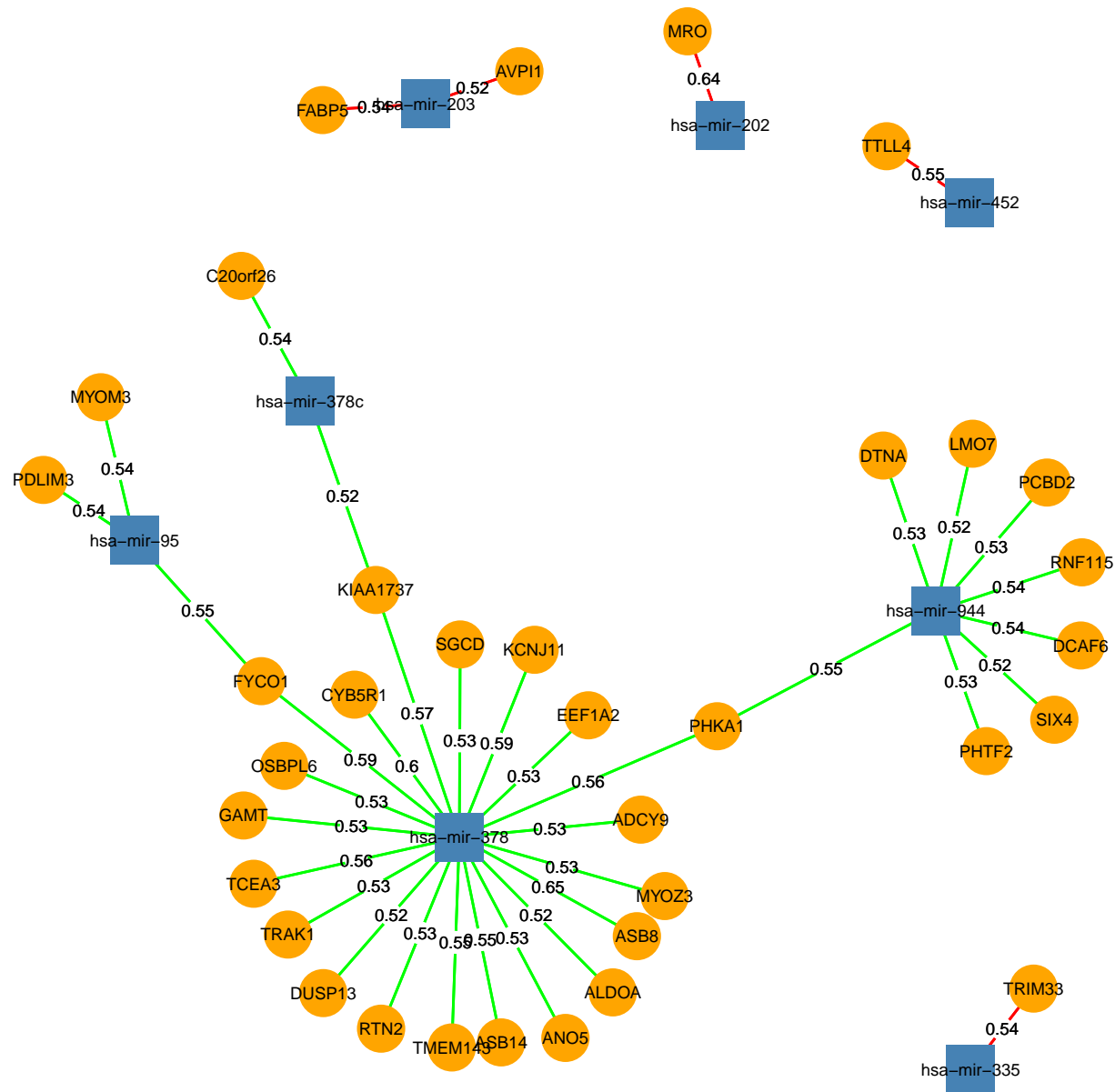

Supplement: Supplementary file 1 — Description of data: top 38 edge-weighted miRNA-mRNA pairs of all six edge weight formulas clustered by traditional hierarchical clustering algorithm are shown in the graphs. (ZIP 39 kb) [file 12920_2019_562_MOESM1_ESM.zip › arithmetic_mean_value_weight.top_38_edges.BRCAR2.pdf]

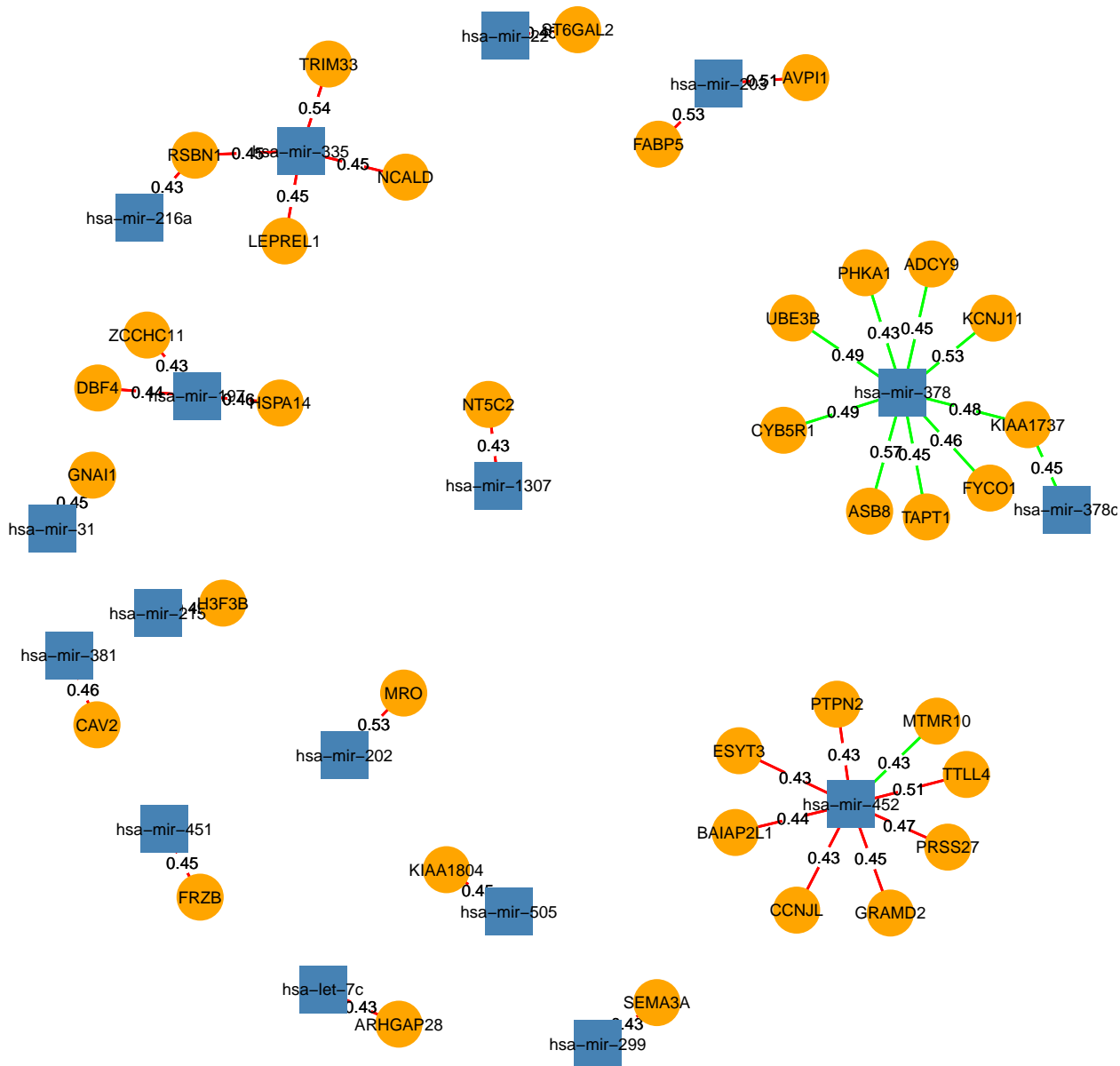

Supplement: Supplementary file 1 — Description of data: top 38 edge-weighted miRNA-mRNA pairs of all six edge weight formulas clustered by traditional hierarchical clustering algorithm are shown in the graphs. (ZIP 39 kb) [file 12920_2019_562_MOESM1_ESM.zip › geometric_mean_value_weight.top_38_edges.BRCAR2.pdf]

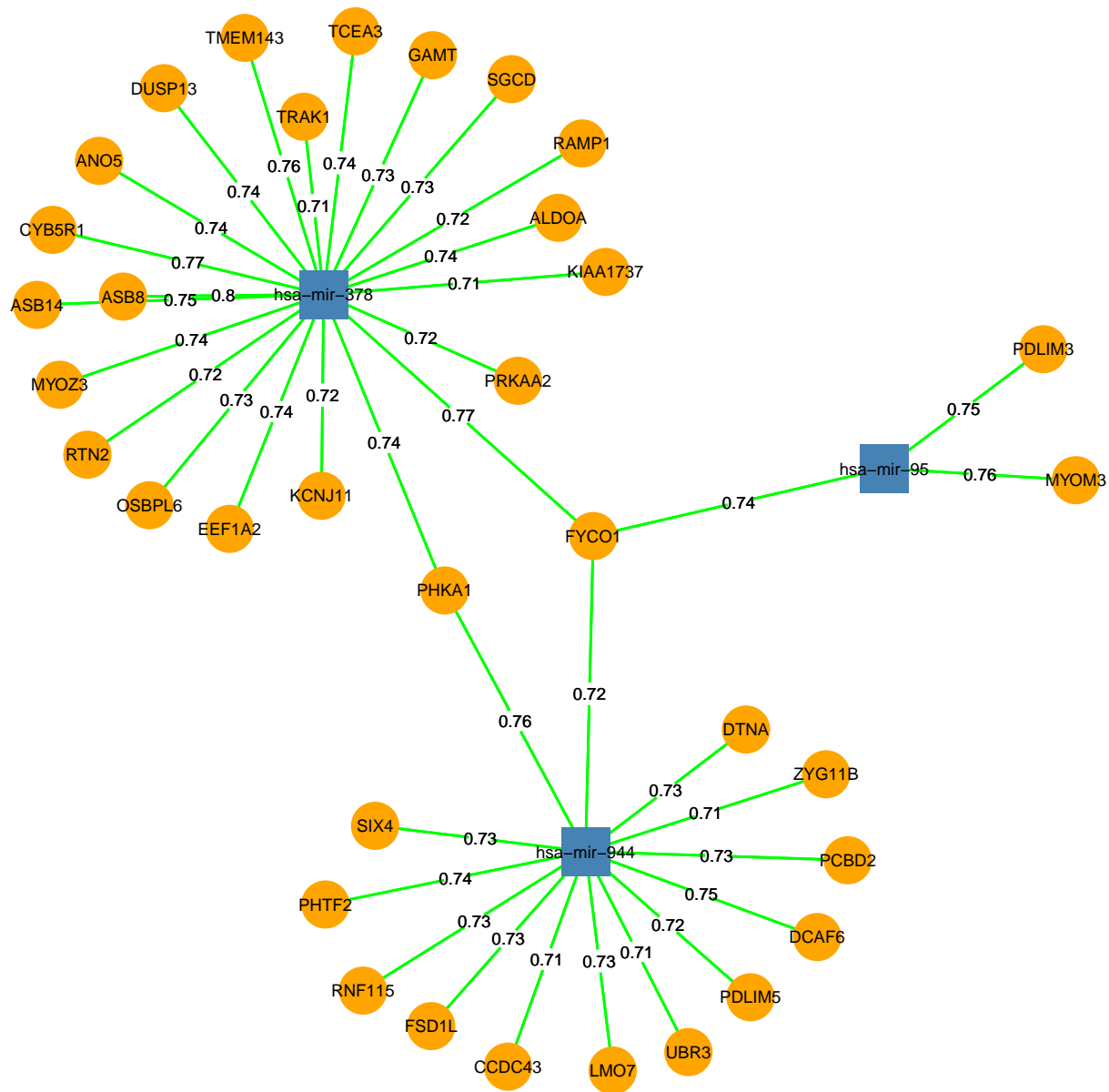

Supplement: Supplementary file 1 — Description of data: top 38 edge-weighted miRNA-mRNA pairs of all six edge weight formulas clustered by traditional hierarchical clustering algorithm are shown in the graphs. (ZIP 39 kb) [file 12920_2019_562_MOESM1_ESM.zip › integrated_mean_value_weight.top_38_edges.BRCAR2.pdf]

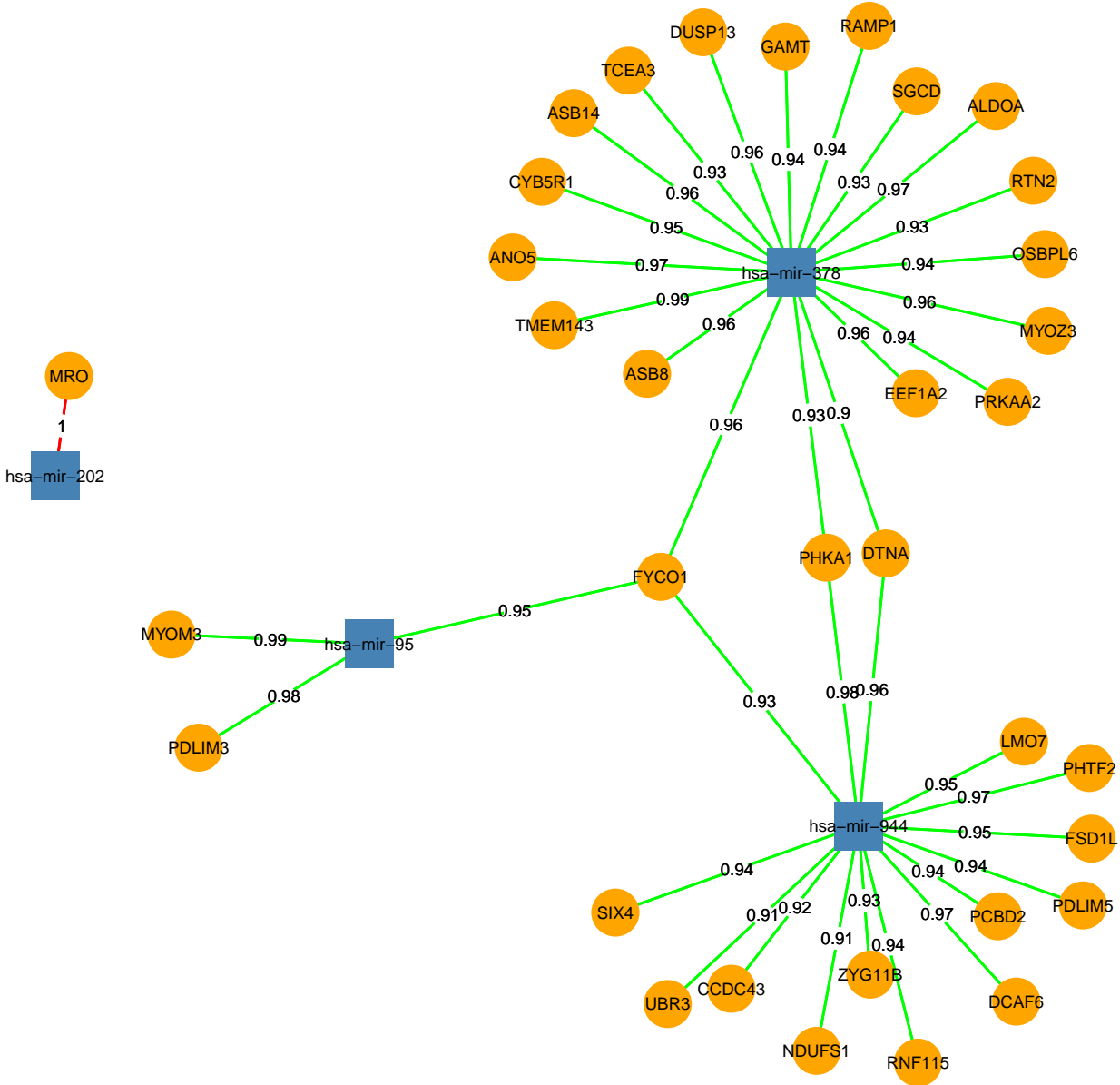

Supplement: Supplementary file 1 — Description of data: top 38 edge-weighted miRNA-mRNA pairs of all six edge weight formulas clustered by traditional hierarchical clustering algorithm are shown in the graphs. (ZIP 39 kb) [file 12920_2019_562_MOESM1_ESM.zip › maximum_absolute_value_weight.top_38_edges.BRCAR2.pdf]

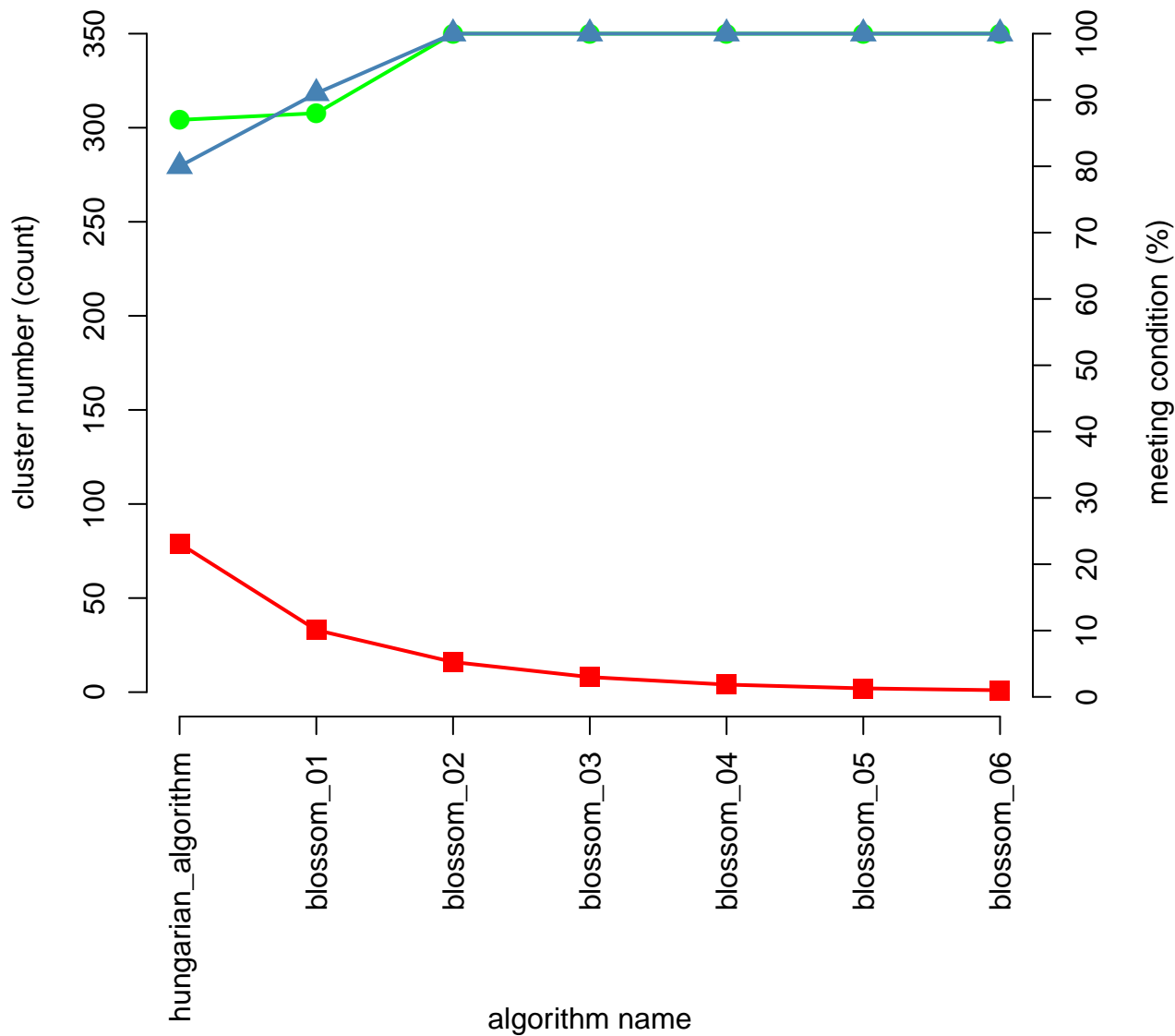

Supplement: Supplementary file 3 — Description of data: the graphs describe the change of cluster numbers and mathematical condition satisfactions as more merging rounds are applied to different cancer types by MWMM approach. The supplementary graphs have the same setting as Figs. 17 and 23 in the context. (ZIP 62 kb) [file 12920_2019_562_MOESM3_ESM.zip › inner_and_outer_weight_conditions.MWMM_only.BLCAR2.pdf]

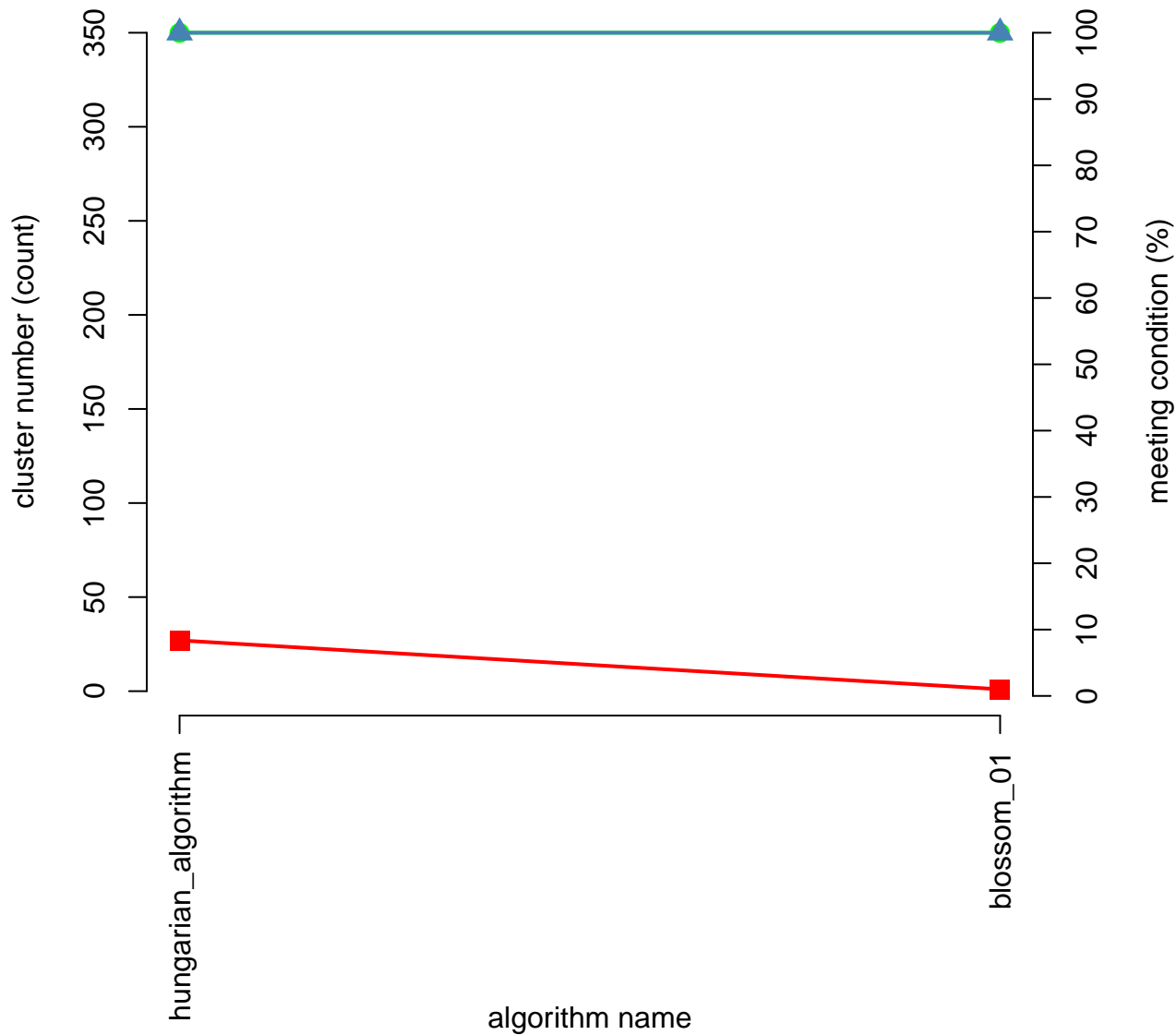

Supplement: Supplementary file 3 — Description of data: the graphs describe the change of cluster numbers and mathematical condition satisfactions as more merging rounds are applied to different cancer types by MWMM approach. The supplementary graphs have the same setting as Figs. 17 and 23 in the context. (ZIP 62 kb) [file 12920_2019_562_MOESM3_ESM.zip › inner_and_outer_weight_conditions.MWMM_only.COADR2.pdf]

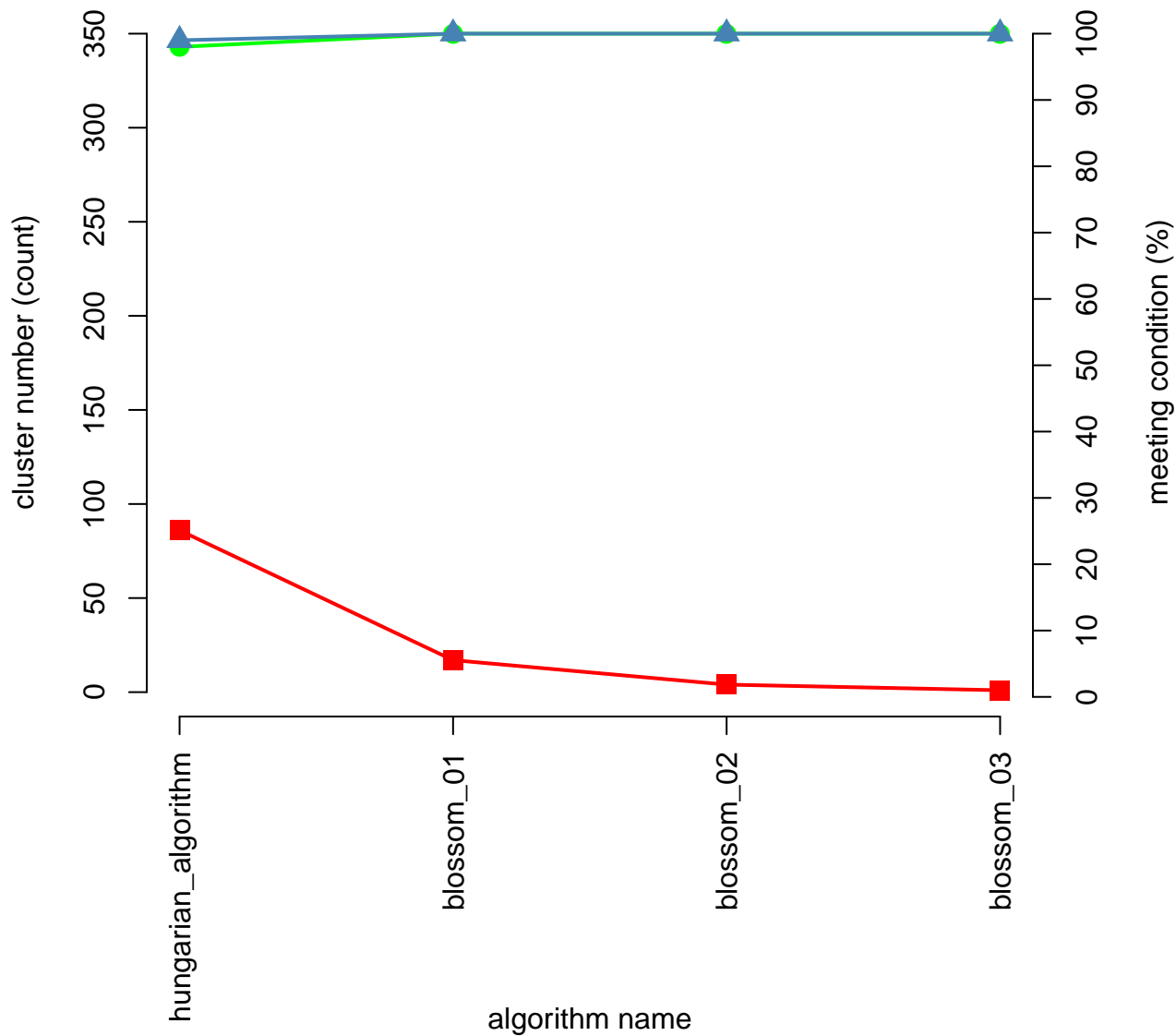

Supplement: Supplementary file 3 — Description of data: the graphs describe the change of cluster numbers and mathematical condition satisfactions as more merging rounds are applied to different cancer types by MWMM approach. The supplementary graphs have the same setting as Figs. 17 and 23 in the context. (ZIP 62 kb) [file 12920_2019_562_MOESM3_ESM.zip › inner_and_outer_weight_conditions.MWMM_only.ESCAR2.pdf]

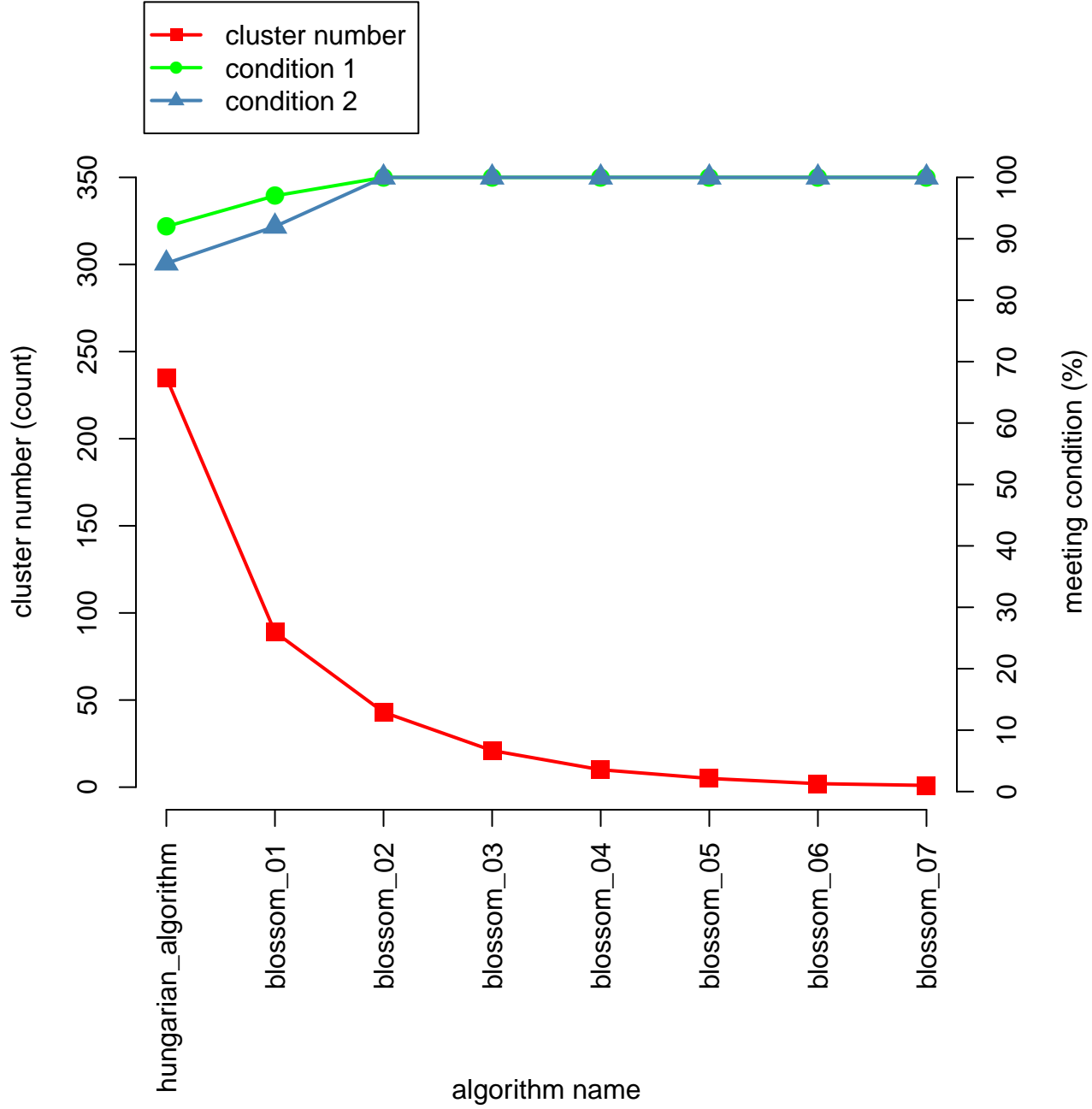

Supplement: Supplementary file 3 — Description of data: the graphs describe the change of cluster numbers and mathematical condition satisfactions as more merging rounds are applied to different cancer types by MWMM approach. The supplementary graphs have the same setting as Figs. 17 and 23 in the context. (ZIP 62 kb) [file 12920_2019_562_MOESM3_ESM.zip › inner_and_outer_weight_conditions.MWMM_only.HNSCR2.pdf]

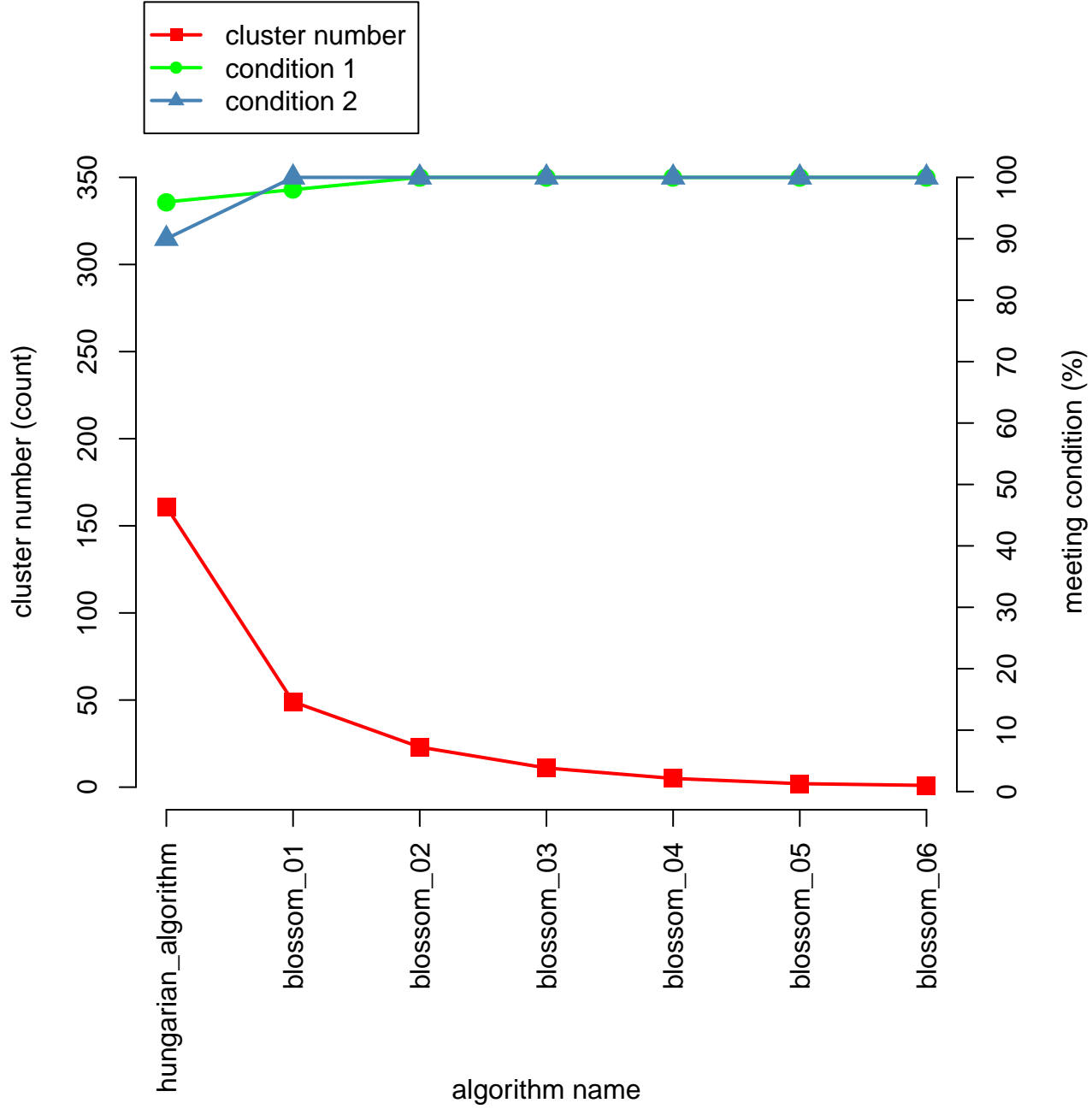

Supplement: Supplementary file 3 — Description of data: the graphs describe the change of cluster numbers and mathematical condition satisfactions as more merging rounds are applied to different cancer types by MWMM approach. The supplementary graphs have the same setting as Figs. 17 and 23 in the context. (ZIP 62 kb) [file 12920_2019_562_MOESM3_ESM.zip › inner_and_outer_weight_conditions.MWMM_only.KICHR2.pdf]

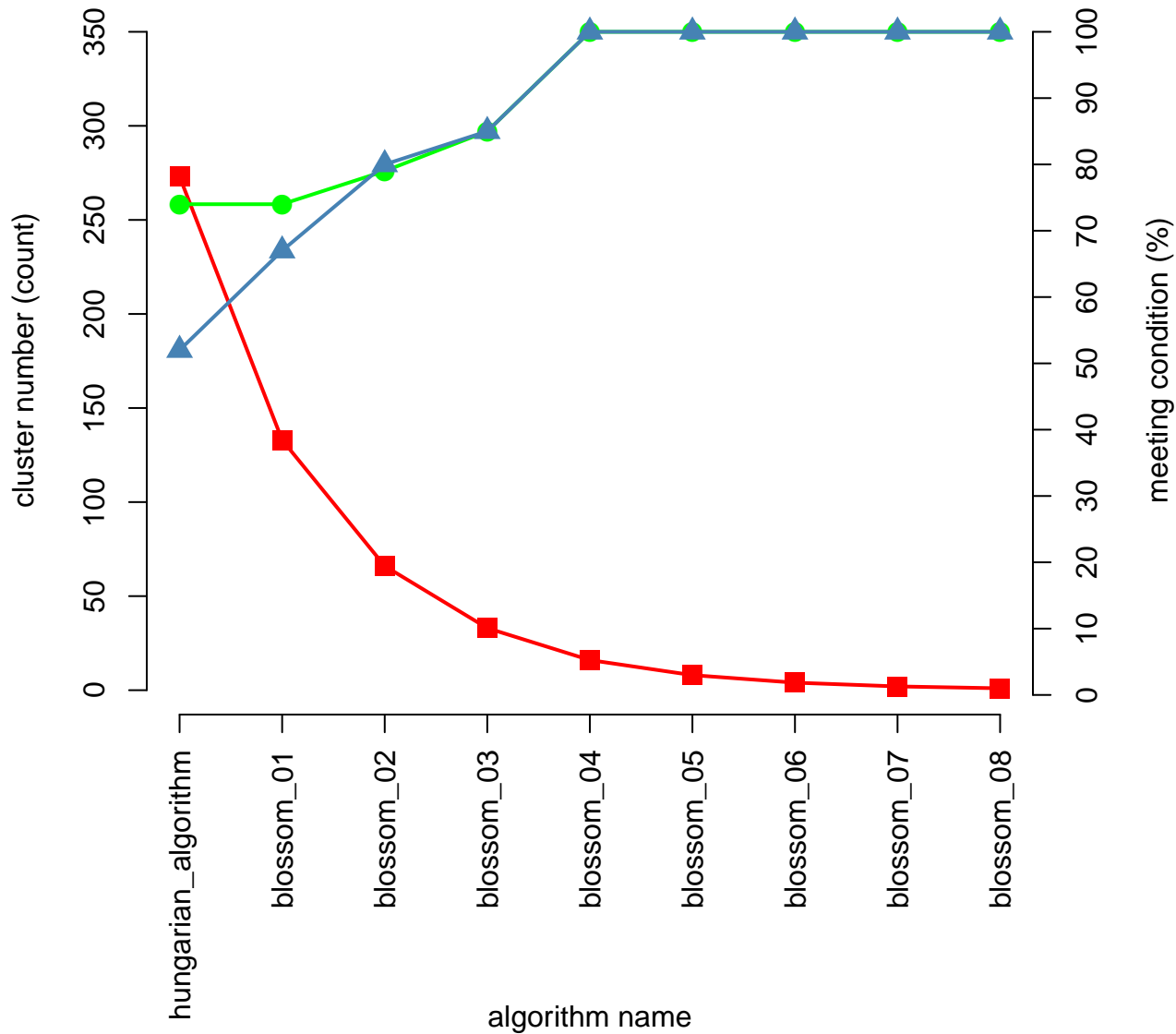

Supplement: Supplementary file 3 — Description of data: the graphs describe the change of cluster numbers and mathematical condition satisfactions as more merging rounds are applied to different cancer types by MWMM approach. The supplementary graphs have the same setting as Figs. 17 and 23 in the context. (ZIP 62 kb) [file 12920_2019_562_MOESM3_ESM.zip › inner_and_outer_weight_conditions.MWMM_only.KIRCR2.pdf]

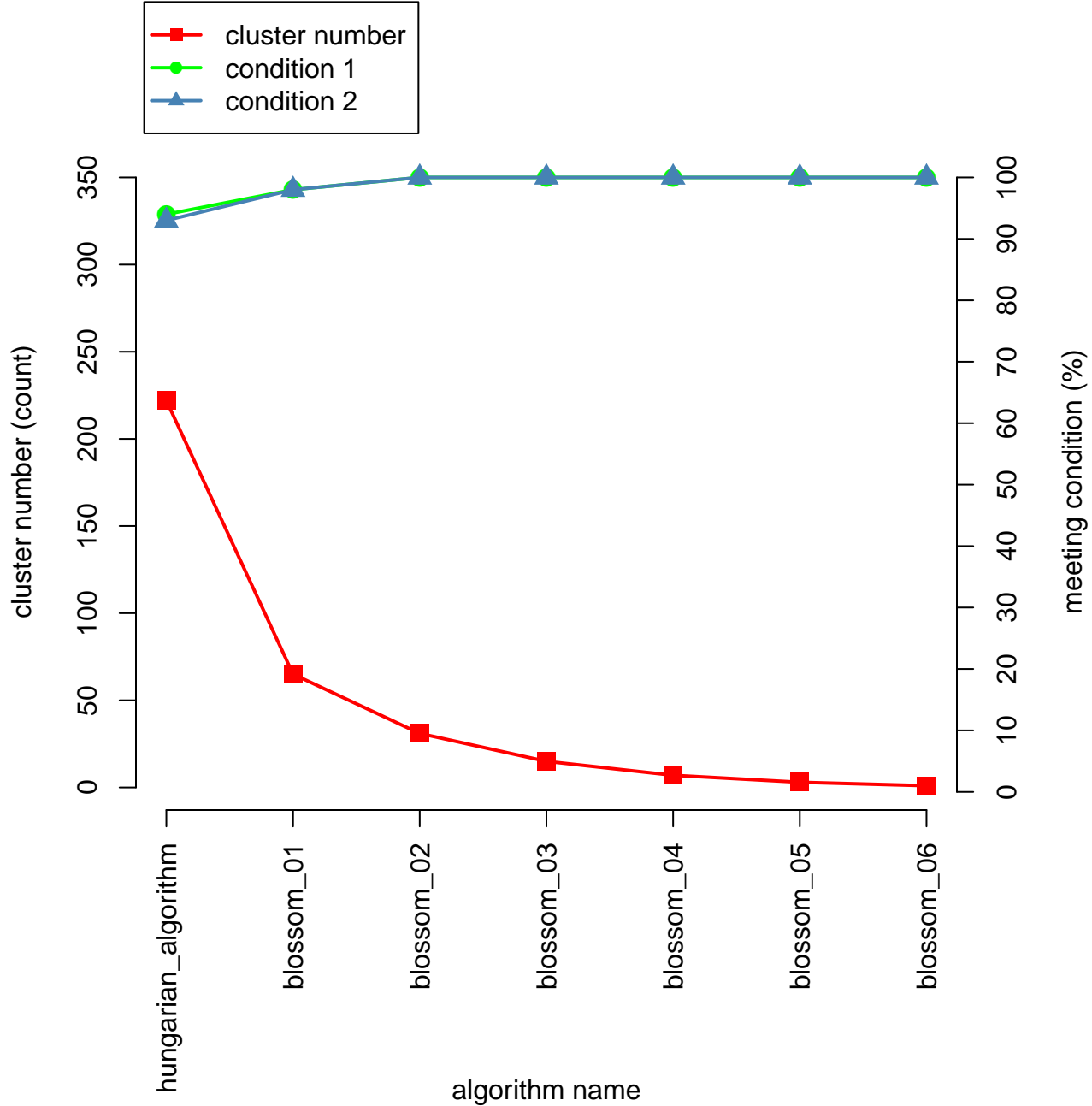

Supplement: Supplementary file 3 — Description of data: the graphs describe the change of cluster numbers and mathematical condition satisfactions as more merging rounds are applied to different cancer types by MWMM approach. The supplementary graphs have the same setting as Figs. 17 and 23 in the context. (ZIP 62 kb) [file 12920_2019_562_MOESM3_ESM.zip › inner_and_outer_weight_conditions.MWMM_only.LIHCR2.pdf]

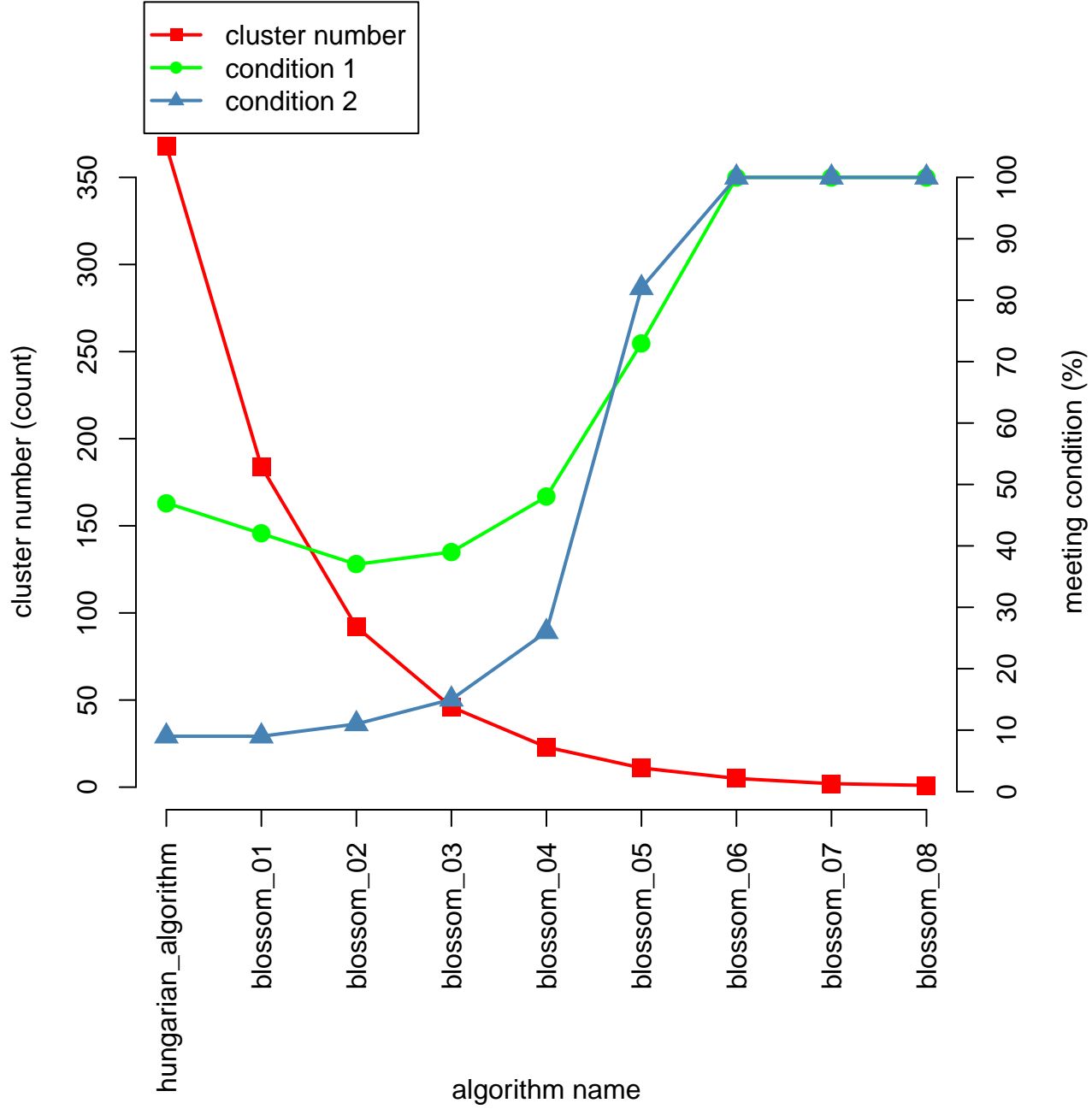

Supplement: Supplementary file 3 — Description of data: the graphs describe the change of cluster numbers and mathematical condition satisfactions as more merging rounds are applied to different cancer types by MWMM approach. The supplementary graphs have the same setting as Figs. 17 and 23 in the context. (ZIP 62 kb) [file 12920_2019_562_MOESM3_ESM.zip › inner_and_outer_weight_conditions.MWMM_only.LUADR2.pdf]

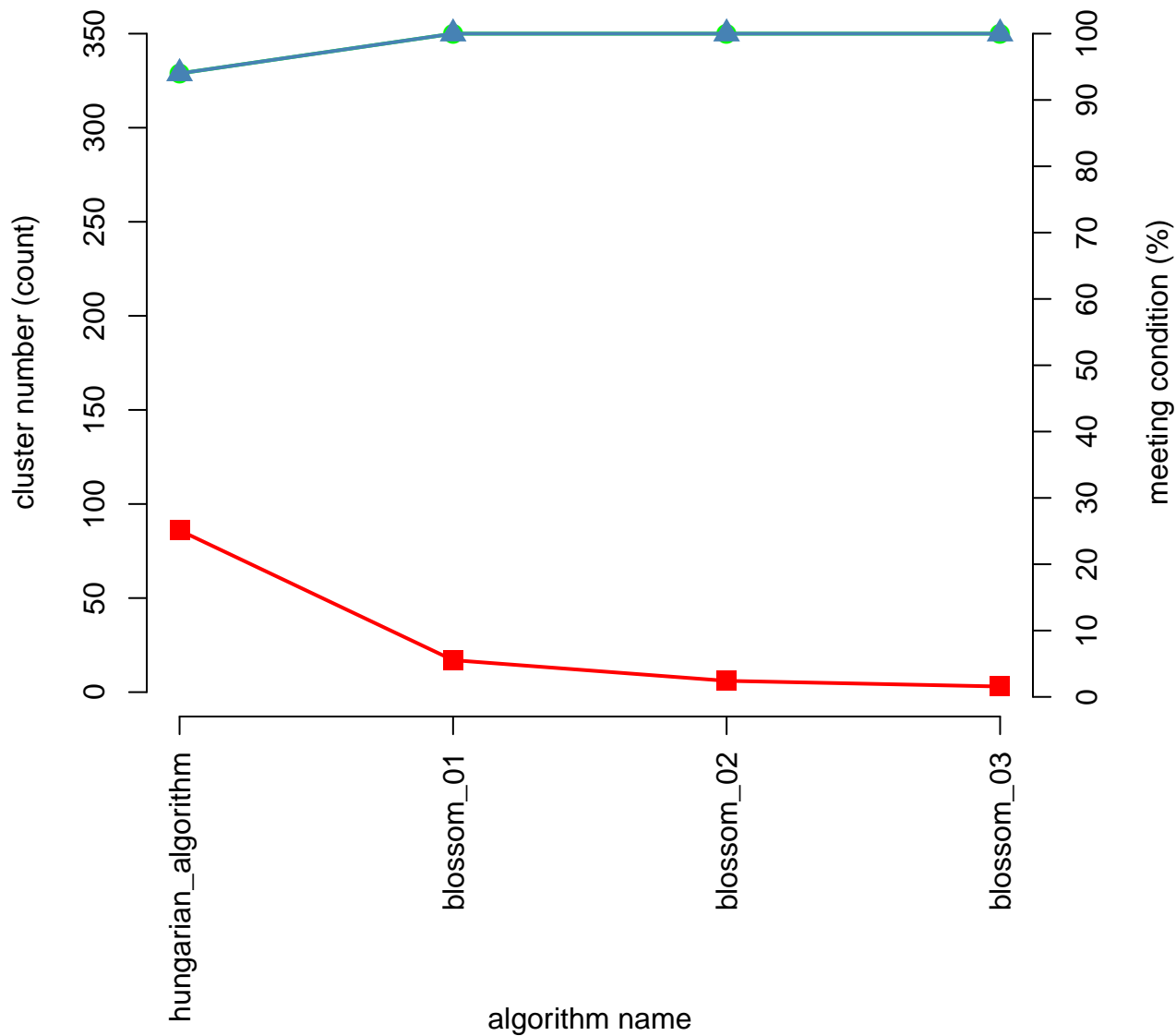

Supplement: Supplementary file 3 — Description of data: the graphs describe the change of cluster numbers and mathematical condition satisfactions as more merging rounds are applied to different cancer types by MWMM approach. The supplementary graphs have the same setting as Figs. 17 and 23 in the context. (ZIP 62 kb) [file 12920_2019_562_MOESM3_ESM.zip › inner_and_outer_weight_conditions.MWMM_only.LUSCR2.pdf]

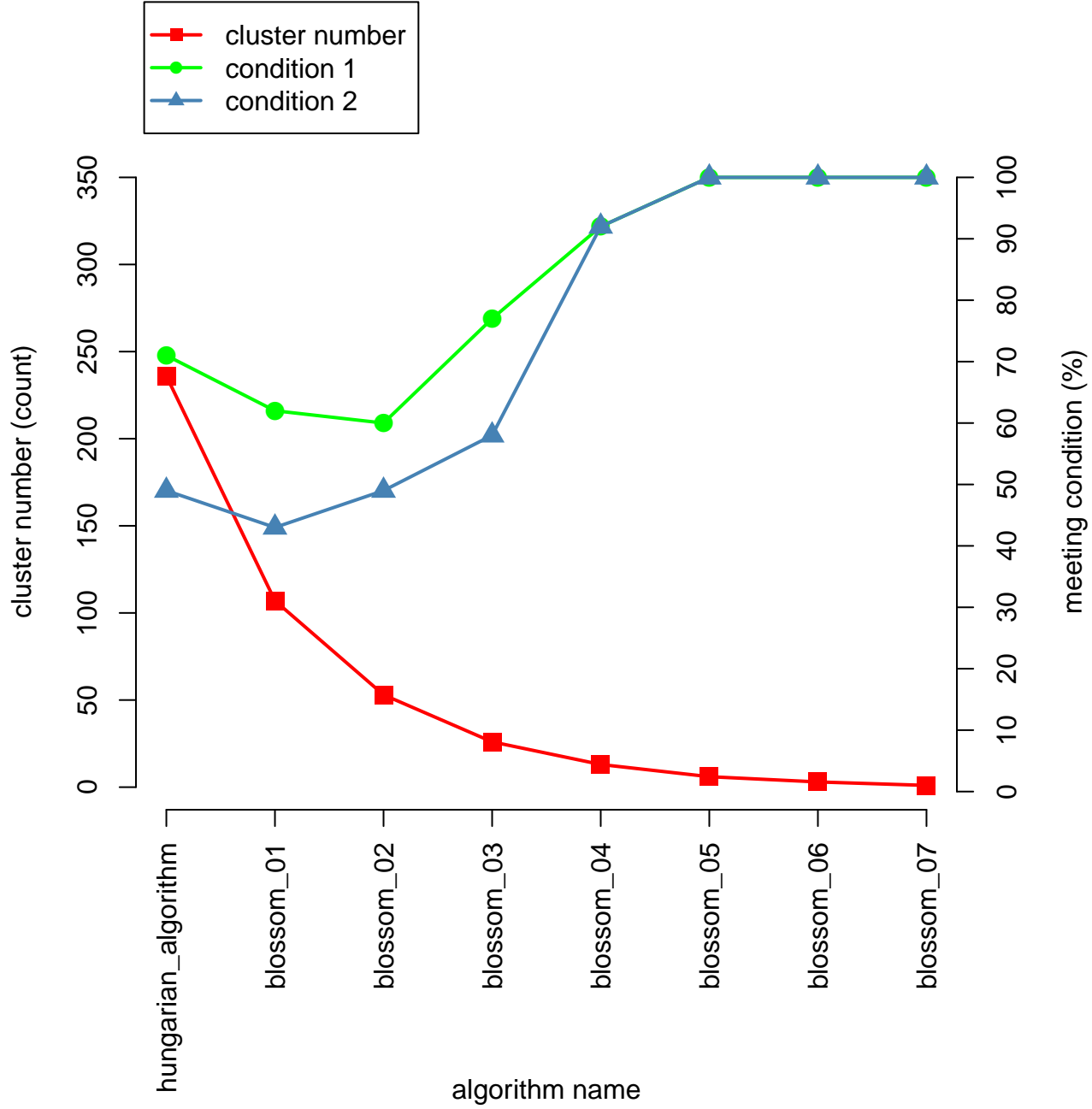

Supplement: Supplementary file 3 — Description of data: the graphs describe the change of cluster numbers and mathematical condition satisfactions as more merging rounds are applied to different cancer types by MWMM approach. The supplementary graphs have the same setting as Figs. 17 and 23 in the context. (ZIP 62 kb) [file 12920_2019_562_MOESM3_ESM.zip › inner_and_outer_weight_conditions.MWMM_only.PRADR2.pdf]

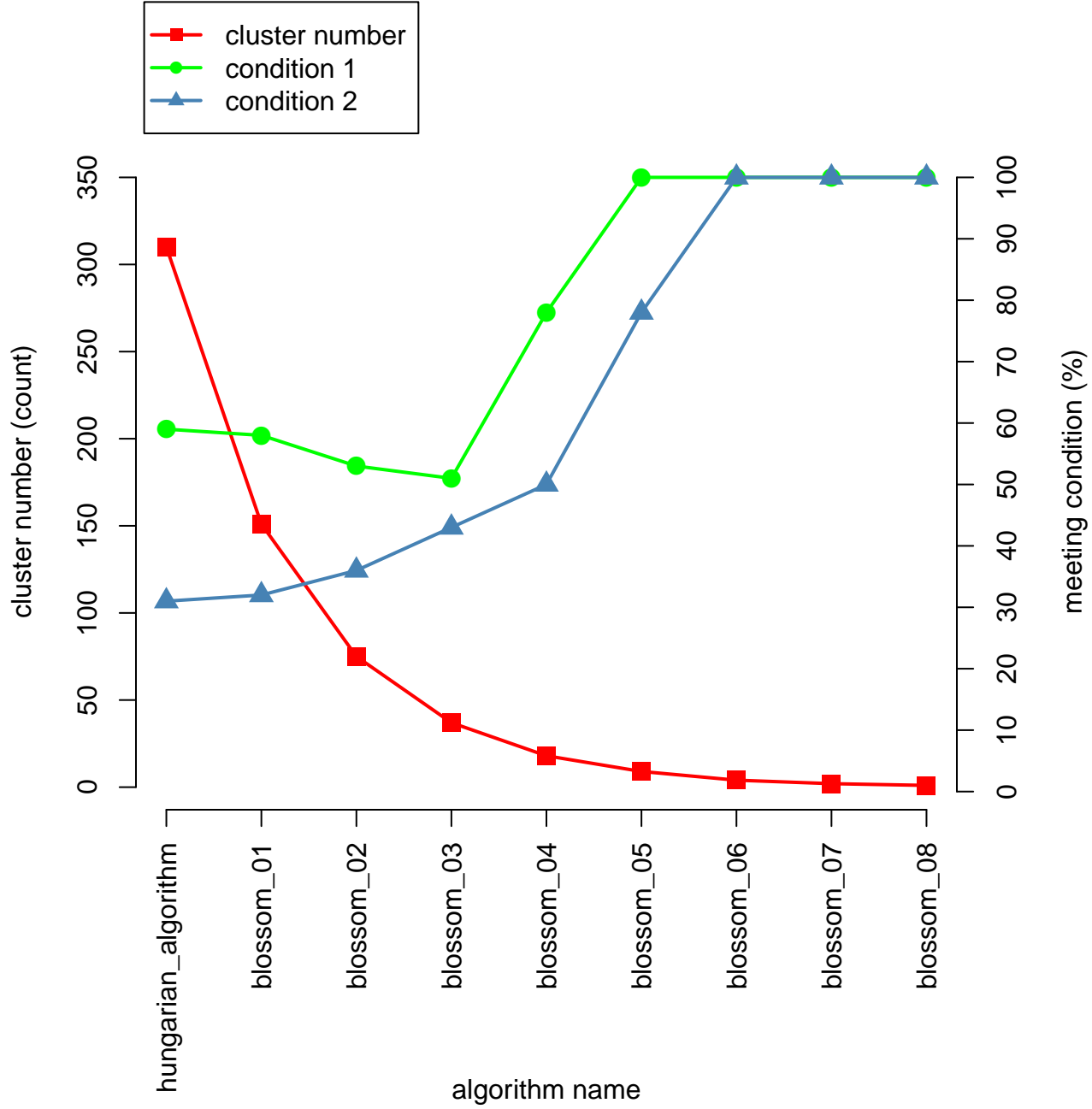

Supplement: Supplementary file 3 — Description of data: the graphs describe the change of cluster numbers and mathematical condition satisfactions as more merging rounds are applied to different cancer types by MWMM approach. The supplementary graphs have the same setting as Figs. 17 and 23 in the context. (ZIP 62 kb) [file 12920_2019_562_MOESM3_ESM.zip › inner_and_outer_weight_conditions.MWMM_only.STADR2.pdf]

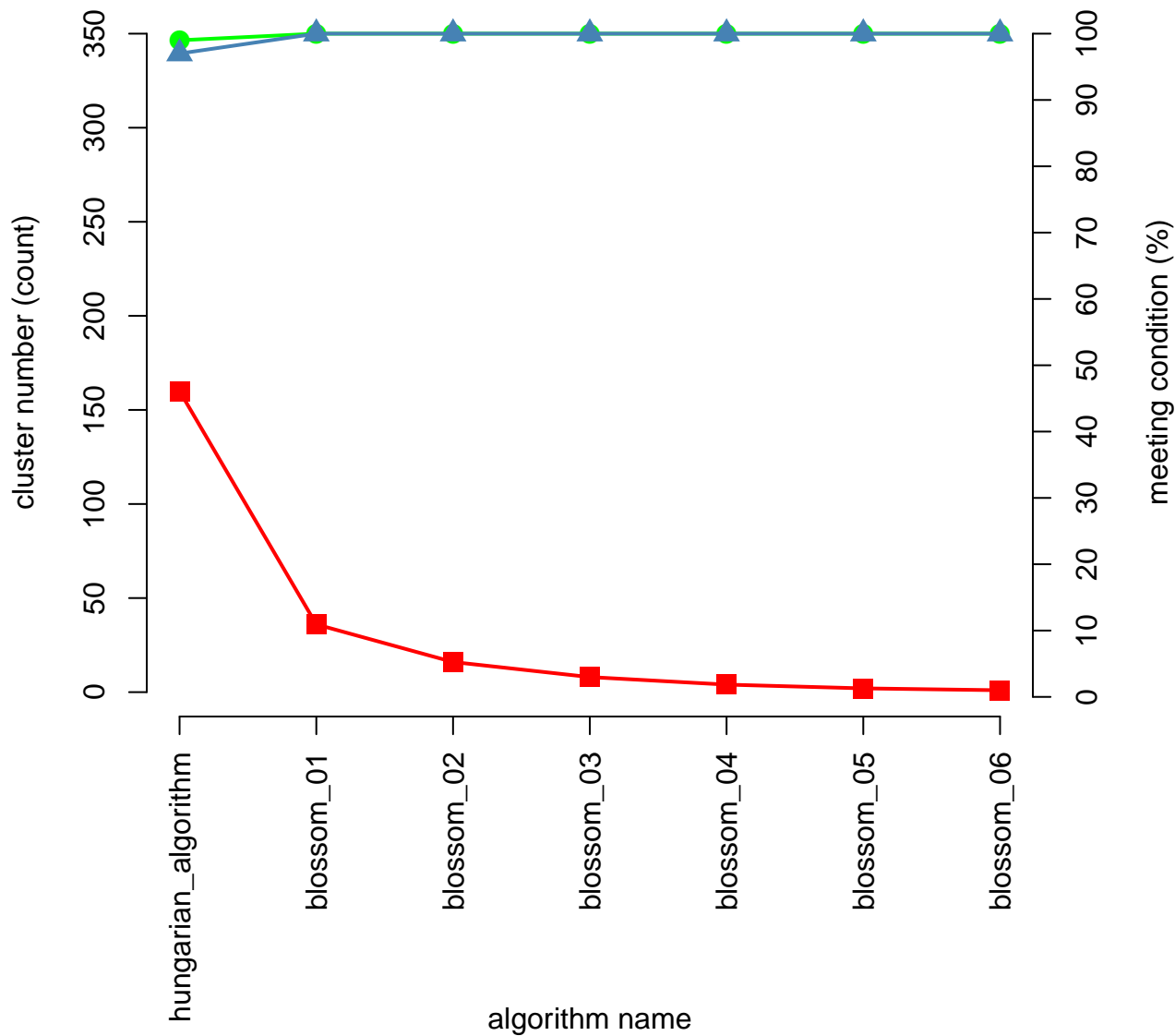

Supplement: Supplementary file 3 — Description of data: the graphs describe the change of cluster numbers and mathematical condition satisfactions as more merging rounds are applied to different cancer types by MWMM approach. The supplementary graphs have the same setting as Figs. 17 and 23 in the context. (ZIP 62 kb) [file 12920_2019_562_MOESM3_ESM.zip › inner_and_outer_weight_conditions.MWMM_only.THCAR2.pdf]

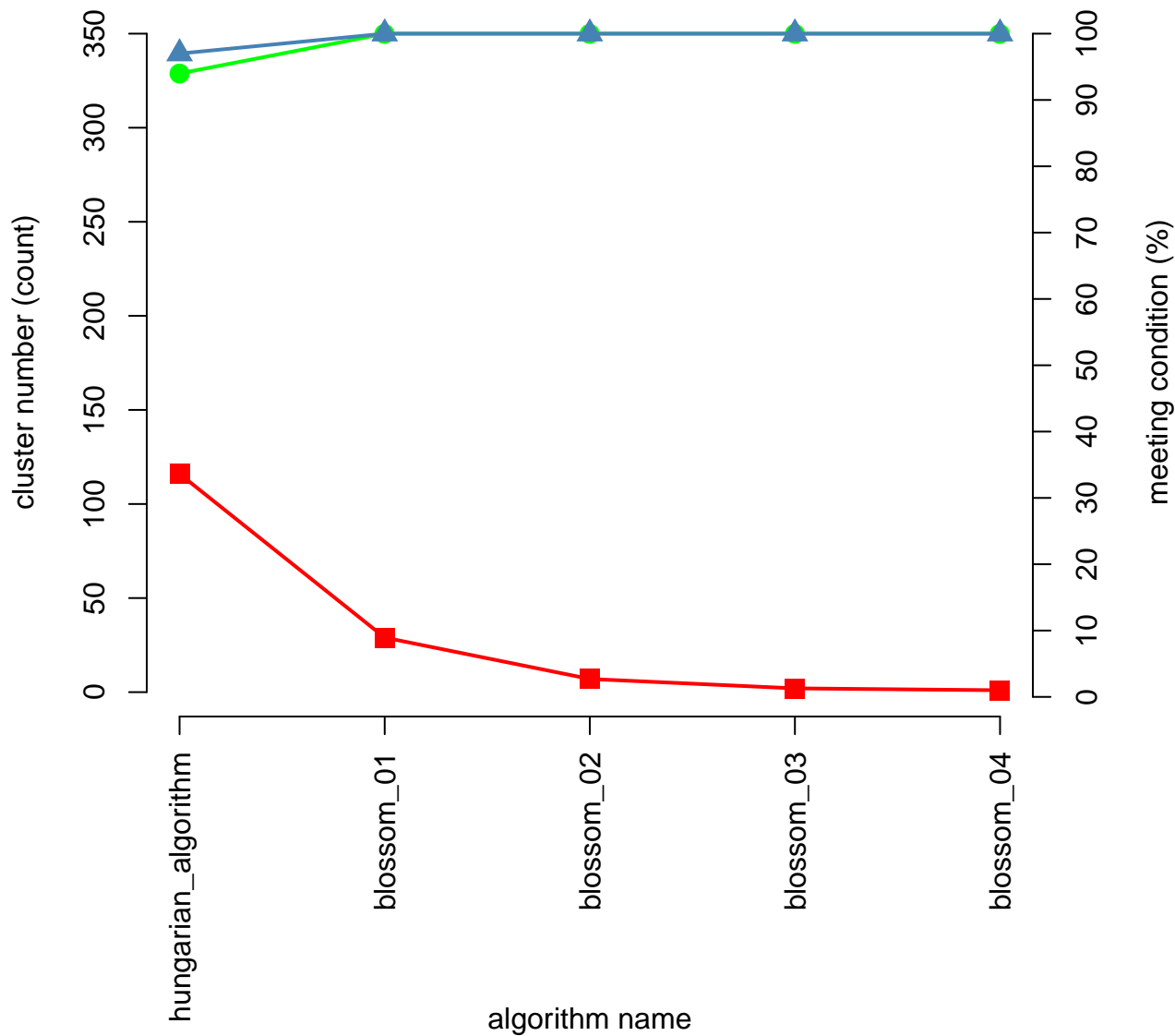

Supplement: Supplementary file 3 — Description of data: the graphs describe the change of cluster numbers and mathematical condition satisfactions as more merging rounds are applied to different cancer types by MWMM approach. The supplementary graphs have the same setting as Figs. 17 and 23 in the context. (ZIP 62 kb) [file 12920_2019_562_MOESM3_ESM.zip › inner_and_outer_weight_conditions.MWMM_only.UCECR2.pdf]
